# Synthesis of the Indole-Based Inhibitors of Bacterial Cystathionine γ-Lyase NL1-NL3

**DOI:** 10.3390/molecules28083568

**Published:** 2023-04-19

**Authors:** Konstantin V. Potapov, Roman A. Novikov, Maxim A. Novikov, Pavel N. Solyev, Yury V. Tomilov, Sergey N. Kochetkov, Alexander A. Makarov, Vladimir A. Mitkevich

**Affiliations:** 1 Engelhardt Institute of Molecular Biology of the Russian Academy of Sciences, 32 Vavilov St., 119991 Moscow, Russiasolyev@gmail.com (P.N.S.);; 2 Zelinsky Institute of Organic Chemistry of the Russian Academy of Sciences, 47 Leninsky Avenue, 119991 Moscow, Russia; 3 Biotechnology Department, Sirius University of Science and Technology, 1 Olympic Avenue, 354349 Sirius, Russia

**Keywords:** antibacterial compounds, indole, bCSE, antibiotics potentiation

## Abstract

Bacterial cystathionine γ-lyase (bCSE) is the main producer of H_2_S in pathogenic bacteria such as *Staphylococcus aureus*, *Pseudomonas aeruginosa*, etc. The suppression of bCSE activity considerably enhances the sensitivity of bacteria to antibiotics. Convenient methods for the efficient synthesis of gram quantities of two selective indole-based bCSE inhibitors, namely (2-(6-bromo-1*H*-indol-1-yl)acetyl)glycine (NL1), 5-((6-bromo-1*H*-indol-1-yl)methyl)- 2-methylfuran-3-carboxylic acid (NL2), as well as a synthetic method for preparation 3-((6-(7-chlorobenzo[*b*]thiophen-2-yl)-1*H*-indol-1-yl)methyl)- 1*H*-pyrazole-5-carboxylic acid (NL3), have been developed. The syntheses are based on the use of 6-bromoindole as the main building block for all three inhibitors (NL1, NL2, and NL3), and the designed residues are assembled at the nitrogen atom of the 6-bromoindole core or by the substitution of the bromine atom in the case of NL3 using Pd-catalyzed cross-coupling. The developed and refined synthetic methods would be significant for the further biological screening of NL-series bCSE inhibitors and their derivatives.

## 1. Introduction

**Figure 1:**
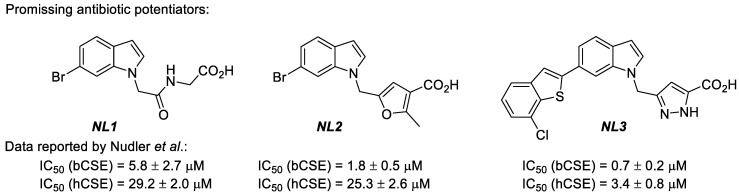
NL1, NL2, and NL3 inhibitors of the bacterial cystathionine γ-lyase (bCSE). IC_50_ data was reported by Nudler et al. [3].

The majority of antibiotics used to treat infectious diseases are aimed at inhibiting essential bacterial proteins. However, the efficiency of traditional antibiotics decreases. At the same time, pathogenic bacteria develop resistance to their action, which makes it necessary to increase the concentrations of antibiotics used for therapy, combine them, and look for new antibacterial drugs. As an alternative approach, the effect of antibiotics that trigger damage in bacteria via oxidative stress can also be enhanced by blocking bacterial H_2_S-producing enzymes and, consequently, the production of H_2_S and glutathione. Quite recently, cystathionine γ-lyase (CSE) was suggested as an additional drug target, provided that selective inhibitors of this enzyme in bacterial pathogens (bCSE) and the low activity of these molecules against the human version of the enzyme (hCSE) are ensured [1]. In many pathogenic bacteria (e.g., *Staphylococcus aureus* or *Pseudomonas aeruginosa*), bCSE is the primary H_2_S producer and is usually involved in forming their resistance mechanisms relative to active antibiotics. In a recent study, Nudler’s team performed large-scale in silico and in vitro screening. It succeeded in identifying three leaders among bCSE inhibitors that featured high activity, selectivity, and low toxicity [2]. These include the NL1 ((2-(6-bromo-1*H*-indol-1-yl)acetyl)glycine), NL2 (5-((6-bromo-1*H*-indol-1-yl)methyl)-2-methylfuran-3-carboxylic acid), and NL3 (3-((6-(7-chlorobenzo[*b*]thiophen-2-yl)-1*H*-indol-1-yl)methyl)-1*H*-pyrazole-5-carboxylic acid) molecules, i.e., derivatives based on 6-bromoindole (Figure 1). It was shown that these compounds could be applied as potentiators for a considerable enhancement of the antibiotic effect on pathogenic bacterial microorganisms, including resistant strains. Thus, the further development of this approach requires efficient and readily available methods for synthesising NL1, NL2, and NL3 inhibitors, which have not been reported on a gram scale to date [3]. Our work aims to develop and optimize methods for synthesising NL1, NL2, and NL3 in near-gram quantities for biological tests with the careful verification of all the products using physicochemical methods.

## 2. Results and Discussion

### 2.1. Synthesis of 6-Bromoindole

**Figure 2:**
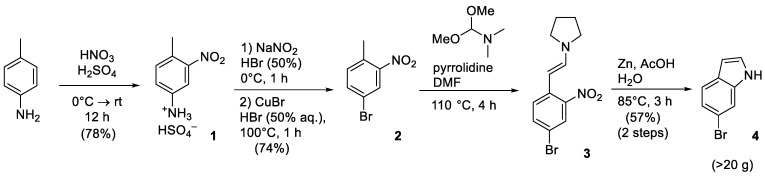
Synthesis of 6-bromoindole.

Since the structures of all three target bCSE inhibitors, i.e., NL1, NL2, and NL3, are based on a fragment of 6-bromoindole or its derivative, the initial step was to choose a convenient synthesis of 6-bromoindole **4** as one of the main building blocks in amounts of at least several tens of grams. About a dozen of methods for its synthesis can be found in the literature [4,5,6]; a complete description of their drawbacks and advantages (along with a synthesis of another important natural derivative, 6-bromotryptamine) was summarized in the recent review article [7]. However, many reported methods possess problems with scalability, the availability of initial reagents, and low yields. From our point of view, the four-stage scheme based on the diazotization of *para*-aminotoluene followed by bromination [8] and the ring closure in two stages (Figure 2) proved to be the most convenient approach [7].

This reaction sequence is characterized by moderate yields; it is easy to optimize and scale up to the required quantities, and it employs inexpensive starting reagents.

Thus, the initial 6-bromoindole **4** was synthesized, and the target inhibitors were subsequently obtained.

### 2.2. Synthesis of NL1 Inhibitor

**Figure 3:**
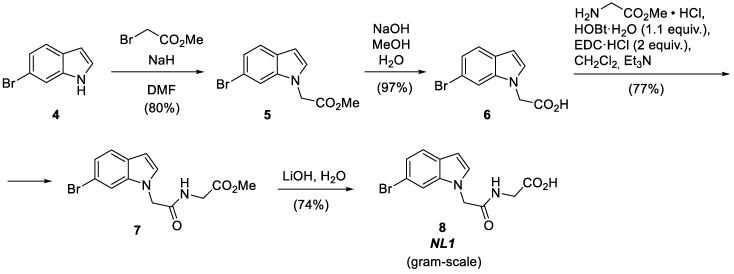
Synthesis of the NL1 inhibitor.

The NL1 molecule is the simplest one in terms of structure and synthesis [2]. NL1 is synthesized from 6-bromoindole in four steps by attaching the short peptide-like chain to the indole nitrogen atom (Figure 3). First, 6-bromoindole **4** is alkylated with bromoacetic ester and then hydrolyzed to yield 2-(6-bromo-1*H*-indol-1-yl) acetic acid **6** [9,10]. The subsequent reaction of the latter with glycine ester hydrochloride under peptide synthesis conditions creates an amide bond [11].

DCC or EDC can be successfully used as a dehydrating agent. However, EDC shows the best results if the reagent ratio is excessive. The hydrolysis of the ester group in the resulting product **7** with lithium hydroxide provides the target compound: NL1 [12]. This synthetic route makes obtaining NL1 in near-gram quantities possible without special additional purification.

### 2.3. Synthesis of NL2 Inhibitor

**Figure 4:**
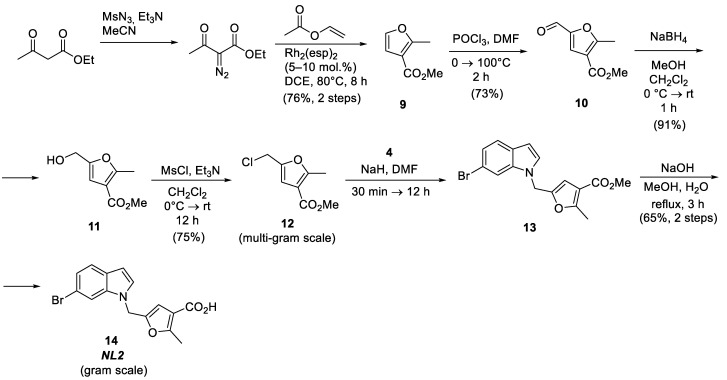
Synthesis of the NL2 inhibitor.

NL2 is one of the most promising bCSE inhibitors [2]. Its structure is based on the 2-methylfuran-3-carboxylic acid derivative in combination with 6-bromoindole linked via a methylene bridge. NL2 is synthesized by coupling these two fragments, followed by the hydrolysis of the ester group in the last two steps (Figure 4). Methyl 5-(chloromethyl)-2-methylfuran-3-carboxylate **12** is obtained in three stages from methyl 2-methylfuran-3-carboxylate **9** [13,14,15] and is used as the furan building block for coupling with 6-bromoindole. Methyl 2-methylfuran-3-carboxylate **9** can be obtained in one step by the rhodium-catalyzed cyclization of vinyl acetate with ethyl diazoacetate [16]. Next, methyl 2-methylfuran-3-carboxylate is introduced into the formylation reaction, followed by the reduction of the aldehyde group with sodium borohydride [13] and the replacement of the hydroxyl by chloride by treatment with mesyl chloride to produce the essential intermediate compound, methyl 5-(chloromethyl)-2-methylfuran-3-carboxylate **12** [15]. This synthetic pathway was significantly optimized during this work to obtain multigram quantities in high yields, which allowed us to obtain the NL2 molecule in near-gram quantities.

### 2.4. Synthesis of NL3 Inhibitor

**Figure 5:**
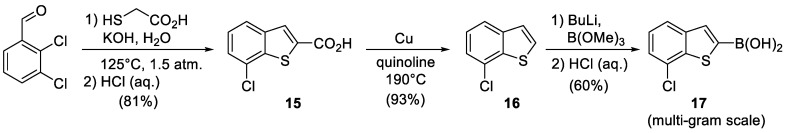
Synthesis of (7-chlorobenzo[*b*]thiophen-2-yl) boronic acid.

NL3, i.e., 3-((6-(7-chlorobenzo[b]thiophen-2-yl)-1*H*-indol-1-yl) methyl)-1*H*-pyrazole-5-carboxylic acid, has the most complex structure of the antibiotic potentiators above. NL3 inhibits bCSE more efficiently than NL2 but is inferior to the latter in selectivity toward hCSE [2]. In contrast to NL1 and NL2, the bromine atom in the indole fragment of NL3 is replaced by 7-chlorobenzo[*b*]thiophene, which can practicably be performed by Pd-catalyzed cross-coupling based on the 6-bromoindole building block that is already developed for NL1 and NL2. The attachment of the heterocyclic fragment by cross-coupling requires that (7-chlorobenzo[*b*]thiophen-2-yl) boronic acid **17** be obtained first. The latter was synthesized from 2,3-dichlorobenzaldehyde in three steps (Figure 5), including the cyclization of the benzothiophene ring, decarboxylation, and subsequent borylation [17,18,19]. The cross-coupling step was performed at the very end of the synthetic sequence after the pyrazole heterocycle had been assembled.

**Figure 6:**
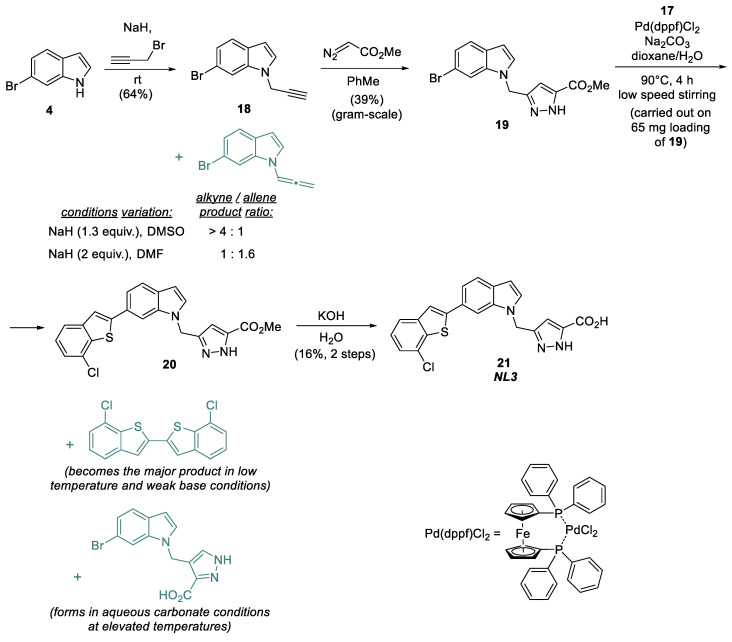
Synthesis of the NL3 inhibitor.

Thus, with **17** as the key reagent, the main part of the NL3 synthetic sequence consists of four steps (Figure 6). 6-Bromoindole **4** is first alkylated with propargyl bromide in the presence of sodium hydride to produce propargylindole **18** [20], which is then introduced into the [3+2]-cycloaddition reaction with ethyl diazoacetate at the triple bond to form the pyrazole ring in product **19** [4].

In the alkylation of bromoindole **4** with propargyl bromide, the excess base reacts with the resulting alkyne **18**. It causes its isomerization to produce the allene as a side product, which is hard to separate from the target propargylindole. To reduce the formation of the undesirable isomer, the base excess should be minimized, and a temperature increase should be avoided; presumably, the yield of the target alkyne can also be increased by using weaker bases that preferably promote NH-activation, such as DBU [21].

The resulting product **19** is introduced into a Pd(dppf)Cl_2_-catalyzed cross-coupling reaction with the previously synthesized (7-chlorobenzo[*b*]thiophen-2-yl) boronic acid **17** under thoroughly selected conditions. The target product NL3 is then easily obtained by the hydrolysis of the ester group, yet the cross-coupling step limits scaling due to the complexity of this stage and the low reactivity of the building blocks being coupled.

We tested several phosphine ligands (XPhos, SPhos, RuPhos, DavePhos, MePhos, CyJohnPhos, PhJohnPhos, XanthPhos, CyXanthPhos, PPh_3_, dppf, DTBPF, and ^t^Bu-XPhos), bases (Na_2_CO_3_, K_2_CO_3_, Cs_2_CO_3_, Na_3_PO_4_, CsF, and ^t^BuONa) and solvents (dioxane/water, toluene, and DMF) [22,23] to improve the cross-coupling step. However, the formation of target product **21** was not observed in any alternative variants. In fact, with weak bases and at room temperature, only the side dimerization of thiopheneboronic acid to produce the dimeric adduct was observed (Figure 6) [24,25]. On the other hand, the reaction using aqueous carbonate bases (Na_2_CO_3_, K_2_CO_3_, and Cs_2_CO_3_) at elevated temperatures mainly resulted in the hydrolysis of **19** relative to the corresponding acid. In contrast, the side reaction of **17** dimerization still occurred. Thus, to date, using Pd(dppf)Cl_2_ as the catalyst and Na_2_CO_3_ as the base is the only option for synthesising compound **20** according to the sequence described above. Moreover, it is important to perform the reaction in a water–dioxane medium at a slow rate by stirring Na_2_CO_3_ at the bottom with a magnetic stirrer. Cross-coupling product **20** is not separable from the original bromoindole **19** by column chromatography. As a result, a mixture of **19** and **20** were used for the hydrolysis, followed by the separation of products by preparative HPLC on the reverse phase C18.

Thus, using the cross-coupling method at the final step of NL3 assembly is the main complicating factor in the synthetic route and requires further studies and optimization, for example, by applying the benzothiophene ring assembly from 6-indole acetic ester under transition-metal-free conditions [26].

### 2.5. Structure Elucidation of NL1, NL2, and NL3

**Figure 7:**
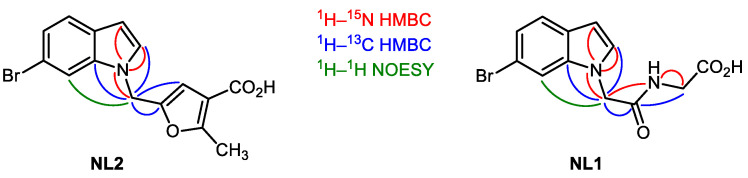
Two-dimensional NMR assignments of the structures.

All compounds synthesized were converted to their lyophilized states, and their structures were confirmed using the standard methods of one- and two-dimensional NMR spectroscopy, including ^15^N experiments and HPLC-HRMS registration. Characteristic correlations confirming the correct cross-linking of fragments were identified in the ^1^H-^1^H, ^1^H-^13^C, and ^1^H-^15^N NMR experiments (Figure 7).

The compounds thus synthesized (NL1, NL2, and NL3) were passed for biological tests.

All NMR spectra are represented in Supplementary Material.

## 3. Conclusions

As a result, we have developed and presented convenient methods for synthesising two efficient bCSE inhibitors, NL1 and NL2, that make it possible to obtain them in gram quantities with a high degree of purity for further biological tests. Unfortunately, during the synthesis of NL3, due to the extremely low yields in the cross-combination reaction, the target substance was obtained only in tens of milligrams. The main synthetic scheme for all inhibitors (NL1, NL2, and NL3) uses 6-bromoindole as the main building block. The rest of the heterocyclic system (pyrazoline, furan, or peptide-like chain) is assembled at the nitrogen atom or with the replacement of the bromine atom by the Pd-catalyzed cross-coupling reaction in the case of NL3. bCSE inhibitors are promising compounds for potentiating antimicrobial therapy and circumventing bacterial resistance. The development of available methods for synthesising these inhibitors would allow their in vivo application modes to be perfected.

## 4. Materials and Methods

### General Experimental Details

All reagents and catalysts were purchased from Sigma-Aldrich, Acros, J&K Scientific and TCI Europe and used without further purification unless otherwise mentioned. TLC analysis was performed on Silufol chromatographic plates. For preparative chromatography, silica gel 60 (0.040–0.063 mm) was used. ^1^H, ^13^C NMR spectra were recorded on a Bruker AVANCE II 300 MHz (300.1, 75.5 MHz and 282.4 MHz, respectively) and a Bruker AMX III 400 MHz (400.1, 100.6 MHz and 376.5 MHz, respectively) spectrometers in CDCl_3_, containing 0.05% Me_4_Si as the internal standard. Determination and verification of structures obtained compounds and assignments of ^1^H and ^13^C signals were made using 1D and 2D DEPT, COSY, HSQC and HMBC spectra. High-resolution mass spectra were recorded on a Bruker Daltonics micrOTOF-Q II device (electrospray ionization). Measurements were carried out in positive ion mode. Samples were injected into the spray chamber of the mass spectrometer from an Agilent 1260 liquid chromatograph equipped with an Agilent Poroshell 120 EC-C18 column (3.0 × 50 mm; 2.7 μm); the flow rate was 0.4 mL min^−1^; the samples of compounds were loaded using autosampler from acetonitrile solution and eluted in the following gradient of acetonitrile (A) in water: 0–6 min—0%–85% A, 6–7.5 min—85% A, 7.5–8 min—85%–0% A, 8–10 min—0% A. Preparative HPLC were performed on Thermo-Finnigan Surveyor equipped with UV-VIS detector on Supelco Ascentis C8 5 μm 250 mm × 10 mm chromatographic column. The starting material 9 was synthesized according to a literature procedure [15].

*4-Bromo-1-methyl-2-nitrobenzene* (**2**), P-toluidine (3.00 g, 28.00 mmol) was added to 6 mL of conc. H_2_SO_4_ when cooled in a water bath (10–15 °C). The water bath was replaced by an ice bath and a mixture of conc. H_2_SO_4_ (6 mL) and conc. HNO_3_ (1.85 g, 29.39 mmol) was added dropwise to the resulting mixture. This solution was stirred at 0 °C for 1 h and another 12 h at rt. The mixture was poured over crushed ice, and the precipitate was filtered, washed with ice-cooled water and dried in high vacuo to afford the intermediate compound **1** as a yellow solid (5.46 g, 78%).

The solution of NaNO_2_ (0.55 g, 7.99 mmol) in 1.3 mL of H_2_O was added to the ice-cooled suspension of aniline hydrosulfate (1.00 g, 3.99 mmol) in 4 mL of H_2_O and 1.3 mL of aq. HBr (50%). The resulting mixture was stirred at 0 °C for 1 h, followed by adding CuBr (0.63 g, 4.40 mmol) solution in 1.3 mL aq. HBr (50%) and 3 mL of H_2_O. The mixture was refluxed for 30 min. After cooling, the mixture was extracted with Et_2_O (3 × 10 mL). The combined organic phases were washed with 10% aq. ammonia, water, dried over MgSO_4_ and concentrated in vacuo. The target compound **2** was crystallized from MeOH as a brown solid (638 mg, 74%)

^1^H NMR (300 MHz, CDCl_3_), δ: 8.13 (d, *J* = 2.1 Hz, 1H), 7.63 (dd, *J* = 8.2, 2.1 Hz, 1H), 7.29–7.20 (m, 1H), 2.57 (s, 3H).^13^C NMR (75 MHz, CDCl_3_), δ: 149.61, 135.96, 134.12, 132.54, 127.53, 119.65, 20.03.

*6-Bromoindole* (**4**), DMF-DMA (1.65 g, 13.89 mmol) and pyrrolidine (0.53 g, 7.41 mmol) were added to the solution of 4-bromo-1-methyl-2-nitrobenzene (1.00 g, 4.63 mmol) in DMF (9 mL). The solution was stirred at 110 °C for 4 h. After cooling, the mixture was dissolved in 20 mL of Et_2_O and washed with 20 mL of H_2_O. Aq. layer was extracted with Et_2_O (3 × 20 mL). The combined organic phases were washed with water and brine, dried over MgSO_4,_ and concentrated in vacuo. Crude intermediate **3** was dissolved in 30 mL of 80% aq. AcOH and heated to 80 °C. Zinc dust (2.42 g, 37.03 mmol) was added to the solution, and the suspension was stirred at this temperature for 3 h. The mixture was cooled down in an ice bath. The precipitate was filtered out and washed with EtOAc. The combined organic phases were washed with water, dried over MgSO_4,_ and concentrated in vacuo. The crude product was purified by silica gel column chromatography (eluent: petroleum ether−AcOEt, 15:1) and by crystallization from petroleum ether to afford the desired compound **4** as a grey solid (513 mg, 57%).

^1^H NMR (300 MHz, CDCl_3_), δ: 8.13 (br.s, 1H), 7.58–7.45 (m, 2H), 7.26–7.12 (m, 2H), 6.53 (ddd, *J* = 3.1, 2.1, 1.0 Hz, 1H).

^13^C NMR (76 MHz, CDCl_3_), δ: 136.60, 126.78, 124.83, 123.18, 121.98, 115.49, 113.99, 102.87.

*Methyl 2-(6-bromo-1H-indol-1-yl)acetate* (**5**), 6-Bromoindole (5.00 g, 25.50 mmol) was added to the stirred suspension of NaH (60% dispersion in oil, 1.53 g, 38.25 mmol) in dry DMF (50 mL). The mixture was stirred for 4 h followed by adding methyl bromoacetate (7.80 g, 51.00 mmol). The resulting mixture was stirred overnight, quenched with 25 mL of H_2_O, and extracted with AcOEt (3 × 25 mL). The combined organic phases were washed with water and brine, dried over MgSO_4,_ and concentrated in vacuo. The crude product was purified by silica gel column chromatography (eluent: petroleum ether−AcOEt, 5:1) to afford the desired compound **5** as a yellow oil (5.50 g, 80%).

^1^H NMR (300 MHz, CDCl_3_), δ: 7.51–7.45 (m, 1H, Ar), 7.39 (dt, *J* = 1.6, 0.6 Hz, 1H, Ar), 7.22 (dd, *J* = 8.4, 1.6 Hz, 1H, Ar), 7.04 (d, *J* = 3.2 Hz, 1H, Ar), 6.52 (dd, *J* = 3.3, 0.9 Hz, 1H, Ar), 4.79 (s, 2H, CH_2_), 3.75 (s, 3H, CH_3_).

^13^C NMR (75 MHz, CDCl_3_), δ: 168.65 (COO), 137.34, 129.12, 127.52, 123.28, 122.37, 115.78, 112.08, 102.84 (Ar), 52.68 (CH_2_), 47.67 (CH_3_).

*2-(6-Bromo-1H-indol-1-yl)acetic acid* (**6**), The solution of NaOH (1.64 g, 41.03 mmol) in water (55 mL) was added to the solution of methyl 2-(6-bromo-1H-indol-1-yl) acetate **5** (5.50 g, 20.52 mmol) in MeOH (55 mL). The mixture was refluxed for 5 h and cooled to room temperature. After that MeOH was evaporated. The aqueous solution was washed with petroleum ether and treated with 10% HCl (aq.) to reach pH = 1. The solution was extracted with AcOEt (3 × 25 mL). The combined organic phases were washed with water and brine, dried over MgSO_4,_ and concentrated in vacuo to make the target compound **6** a colorless solid (5.06 mg, 97%).

^1^H NMR (300 MHz, DMSO-d6), δ: 12.95 (br.s, 1H, OH)7.76–7.63 (m, 1H, Ar), 7.51 (d, *J* = 8.4 Hz, 1H, Ar), 7.36 (d, *J* = 3.2 Hz, 1H, Ar), 7.16 (dd, *J* = 8.4, 1.8 Hz, 1H, Ar), 6.48 (dd, *J* = 3.2, 0.9 Hz, 1H, Ar), 5.05 (s, 2H, CH_2_).

^13^C NMR (75 MHz, DMSO-d6), δ: 170.75 (COO), 137.81, 131.21, 127.55, 122.47, 114.57, 113.27, 101.79 (Ar), 47.58 (CH_2_).

*Methyl 2-(2-(6-bromo-1H-indol-1-yl)acetamido)acetate* (**7**), Glycine methyl ester hydrochloride (2.75 g, 21.92 mmol), hydroxybenzotriazole (hydrate, 80% HOBt, 3.70 g, 21.92 mmol) was added to the solution of 2-(6-bromo-1H-indol-1-yl)acetic acid **6** (5.06 g, 19.95 mmol) in CH_2_Cl_2_ (500 mL), then 1-ethyl-3-(dimethylaminopropyl)carbodiimide hydrochloride (8.77 g, 45.70 mmol) and triethylamine (10.07 mg, 99.53 mmol) were added to the reaction mixture. The mixture was stirred overnight. The solution was treated with 3% HCl (aq.) and extracted with CH_2_Cl_2_. The combined organic layers were washed with H_2_O and brine, dried over Na_2_SO_4,_ and evaporated in vacuo. The product was purified by crystallization from methanol to afford the target compound **7** as a pink powder (4.99 mg, 77%).

^1^H NMR (300 MHz, CDCl_3_), δ: 7.58–7.43 (m, 2H, Ar), 7.37–7.21 (m, 1H, Ar), 7.09 (d, *J* = 3.2 Hz, 1H, Ar), 6.60 (dd, *J* = 3.2, 0.9 Hz, 1H, Ar), 5.88 (br.s, 1H, NH), 4.78 (s, 2H, CH_2_), 3.95 (d, *J* = 5.5 Hz, 2H, CH_2_), 3.69 (s, 3H, CH_3_).

^13^C NMR (75 MHz, CDCl_3_), δ: 169.52 (COO), 168.10 (COO), 137.09, 128.86, 127.67, 123.90, 122.59, 116.34, 112.39, 103.88 (Ar), 52.49 (CH_2_), 49.76 (CH_2_), 40.99 (CH_3_).

*2-(2-(6-Bromo-1H-indol-1-yl)acetamido)acetic acid* (**8**), Lithium hydroxide hydrate was added to the suspension of methyl 2-(2-(6-bromo-1H-indol-1-yl) acetamido) acetate **7** (4.99 g, 15.38 mmol) in THF (25 mL) and water (6 mL). The mixture was stirred overnight and treated with 10% HCl (aq.). The precipitate was filtered, washed with water, and dried in high vacuo. The crude product was crystallized from MeOH to afford the target compound as a pink solid (3.50 g, 74%).

^1^H NMR (300 MHz, DMSO-d6), δ: 12.66 (s, 1H, OH), 8.50 (t, *J* = 5.9 Hz, 1H), 7.68–7.61 (m, 1H), 7.51 (d, *J* = 8.4 Hz, 1H), 7.35 (d, *J* = 3.2 Hz, 1H), 7.16 (dd, *J* = 8.4, 1.8 Hz, 1H), 6.48 (dd, *J* = 3.1, 0.9 Hz, 1H), 4.91 (s, 2H, CH_2_), 3.82 (d, *J* = 5.8 Hz, 2H, CH_2_).

^13^C NMR (75 MHz, DMSO-d6), δ: 171.42, 168.19, 137.69, 131.28, 127.63, 122.47, 114.53, 113.28, 101.65, 48.98, 41.22.

HRMS of C_12_H_11_^79^BrN_2_O_3_ and C_12_H_11_^81^BrN_2_O_3_, *m*/*z*: calcd. for [M + H]^+^ 311.0026 and 313.0006; found 311.0015 and 312.9999.

*Methyl 5-formyl-2-methylfuran-3-carboxylate* (**10**), POCl_3_ (5.5 g, 35.7 mmol) was carefully added to ice-cooled DMF (5 mL). The mixture was heated to room temperature and stirred for 1 h, followed by adding methyl 2-methylfuran-3-carboxylate (5.0 g, 35.7 mmol). The reaction mixture was stirred for another 2 h at 100 °C. The mixture was cooled down to 0 °C, quenched with an aqueous solution of Na_2_CO_3_ (5 g/50 mL H_2_O) and extracted with CH_2_Cl_2_ (3 × 50 mL). The combined organic phases were washed with water and brine, dried over MgSO_4,_ and concentrated in vacuo. The residue was crystallized from Et_2_O. The target compound **10** was collected as a yellow solid (4.3 g, 73%)

^1^H NMR (300 MHz, CDCl_3_), δ: 9.57 (s, 1H, CHO), 7.48 (s, 1H, CH), 3.88 (s, 3H, OCH_3_), 2.70 (s, 3H, CH_3_).

^13^C NMR (75 MHz, CDCl_3_), δ: 177.12, 123.28, 122.30, 114.32, 112.30, 51.88, 14.34.

*Methyl 5-(hydroxymethyl)-2-methylfuran-3-carboxylate* (**11**), NaBH_4_ (1.16 g, 30.64 mmol) was added to the ice-cooled solution of methyl 5-formyl-2-methylfuran-3-carboxylate **10** (4.30 g, 25.59 mmol) in MeOH (70 mL) and CH_2_Cl_2_ (35 mL). The mixture was stirred at 0 °C for 30 min and at room temperature for another 2 h. The resulting solution was quenched with water and extracted with CH_2_Cl_2_. The combined organic phases were washed with water and brine, dried over MgSO_4,_ and concentrated in vacuo. The crude product was purified by silica gel column chromatography (eluent: petroleum ether−AcOEt, 2:1) to afford the target compound **11** as colorless oil (3.93 g, 91%).

^1^H NMR (300 MHz, CDCl_3_), δ: 6.52 (s, 1H, CH), 4.54 (s, 2H, CH_2_), 3.81 (s, 3H, OCH_3_), 2.56 (s, 3H, CH_3_), 2.02 (br.s, 1H, OH).

^13^C NMR (76 MHz, CDCl_3_), δ: 164.57, 159.42, 152.04, 113.77, 108.49, 56.96, 51.36, 13.74.

HRMS of C_8_H_10_O_4_, *m*/*z*: calcd. for [M+H]^+^ 153.0546; found 153.0549.

*Methyl 5-(chloromethyl)-2-methylfuran-3-***carboxylate** (**12**), Mesyl chloride (5.30 g, 46.31 mmol) was added dropwise to the solution of methyl 5-(hydroxymethyl)-2-methylfuran-3-carboxylate **11** (3.93 g, 23.11 mmol) and Et_3_N (4.68 g, 46.31 mmol) in CH_2_Cl_2_ (40 mL). The solution was stirred for 4 h, quenched with water and extracted with CH_2_Cl_2_. The combined organic phases were washed with water and brine, dried over MgSO_4,_ and concentrated in vacuo. The crude product was purified by silica gel column chromatography (eluent: petroleum ether−AcOEt, 10:1) to afford the target compound **12** as colorless oil (3.27 g, 75%).

^1^H NMR (300 MHz, CDCl_3_), δ: 6.61 (s, 1H, CH), 4.52 (s, 2H, CH_2_), 3.82 (s, 3H, OCH_3_), 2.59 (s, 3H, CH_3_).

^13^C NMR (75 MHz, CDCl_3_), δ: 163.98, 160.23, 148.01, 114.33, 110.53, 51.39, 37.13, 13.81.

*Methyl 5-((6-bromo-1H-indol-1-yl)methyl)-2-methylfuran-3-carboxylate* (**13**), 6-Bromoindole (2.72 g, 13.88 mmol) was added to the stirred suspension of NaH (60% dispersion in oil, 610.7 mg, 12.23 mmol) in dry DMF (15 mL). The mixture was stirred for 4 h followed by adding methyl 5-(chloromethyl)-2-methylfuran-3-carboxylate **12** (3.27 g, 17.33 mmol). The solution was heated to 40 °C and stirred at this temperature for 3 h. The mixture was quenched with H_2_O and extracted with AcOEt. The combined organic phases were washed with water and brine, dried over MgSO_4,_ and concentrated in vacuo. The crude product was purified by silica gel column chromatography (eluent: petroleum ether−AcOEt, 5:1) to afford the target compound **13** as pale-yellow oil (3.68 g, 76%).

^1^H NMR (300 MHz, CDCl_3_), δ: 7.51 (dt, *J* = 1.5, 0.6 Hz, 1H, Ar), 7.43 (dd, *J* = 8.4, 0.6 Hz, 1H, Ar), 7.21–7.15 (m, 1H, Ar), 7.04 (d, *J* = 3.2 Hz, 1H), 6.45 (dd, *J* = 3.2, 0.9 Hz, 1H), 6.43 (s, 1H, CH(4)), 5.02 (s, 2H, CH_2_), 3.75 (s, 3H, OCH_3_), 2.47 (s, 3H, CH_3_).

^13^C NMR (75 MHz, CDCl_3_), δ: 164.16, 159.66, 147.79, 136.83, 128.36, 127.60, 123.07, 122.29, 115.55, 114.00 112.42, 109.16, 102.35, 51.41, 42.91, 13.81.

*5-((6-Bromo-1H-indol-1-yl)methyl)-2-methylfuran-3-carboxylic acid* (**14**), The solution of NaOH (846.2 mg, 21.23 mmol) in water (30 mL) was added to the solution of methyl 5-((6-bromo-1H-indol-1-yl) methyl)-2-methylfuran-3-carboxylate **13** (3.68 g, 10.47 mmol) in 30 mL of MeOH. The mixture was refluxed for 3 h and cooled to room temperature. After that MeOH was evaporated. The aqueous solution was treated with 10% HCl (aq.) to reach pH = 1. The precipitate was filtered out, washed with water, and dried in high vacuo to afford the target compound **14** as a colorless solid (2.17 g, 62%).

^1^H NMR (400 MHz, CDCl_3_), δ: 11.46 (br.s, 1H, OH), 7.52–7.48 (m, 1H), 7.43 (d, *J* = 8.4 Hz, 1H, Ar), 7.18 (dd, *J* = 8.4, 1.7 Hz, 1H, Ar), 7.05–7.00 (m, 1H, Ar), 6.47–6.40 (m, 2H, Ar), 5.02 (s, 2H, CH_2_), 2.43 (s, 3H, CH_3_).

^13^C NMR (101 MHz, CDCl_3_), δ: 169.71 (COOH), 160.95, 147.91, 136.79, 128.27, 127.57, 123.07, 122.28, 115.55, 114.22, 112.37, 109.38, 102.37 (Ar), 42.77 (CH_2_), 13.87 (CH_3_).

HRMS of C_15_H_12_^79^BrNO_3_ and C_15_H_12_^81^BrNO_3_, *m*/*z*: calcd. for [M + H]^+^ 334.0073 and 336.0053; found 334.0072 and 336.0058.

*7-Chlorobenzo[b]thiophene-2-carboxylic acid* (**15**), Potassium hydroxide solution (1.16 g, 20.74 mmol) in water (10 mL) was placed in a glass ampoule, and thioglycolic acid (868 mg, 9.43 mmol) was slowly added. 2,3-Dichlorobenzaldehyde (1.65 g, 9.43 mmol) was added to the reaction mixture. The ampoule was soldered, heated to 125 °C and kept under intense stirring for 2 h. The mixture was cooled to room temperature. The precipitate was dissolved in water. The water layer was washed with Et_2_O and acidified with 10% HCl (aq.). The precipitate was filtered, washed with water, and dried in high vacuo to afford the target compound **15** as a colorless powder (1.62 g, 81%).

^1^H NMR (300 MHz, DMSO-d6), δ: 8.20 (s, 1H), 8.01 (dd, *J* = 7.8, 1.0 Hz, 1H), 7.64 (dd, *J* = 7.8, 1.0 Hz, 1H), 7.51 (t, *J* = 7.8 Hz, 1H).

^13^C NMR (75 MHz, DMSO-d6), δ: 163.56, 140.69, 140.43, 136.20, 131.48, 127.29, 125.33.

*7-Chlorobenzo[b]thiophene* (**16**), 7-Chlorobenzothiophene-2-carboxylic acid **15** (500 mg, 2.35 mmol) was dissolved in 5 mL of quinoline. Copper powder (164 mg, 2.58 mmol) was added to the solution. The suspension was heated to 190 °C and kept under intensive stirring for 3 h. The reaction mixture was cooled to room temperature, and 20 mL of 10% HCl (aq.) solution was added. The resulting system was extracted with CH_2_Cl_2_ (3 × 20 mL). The combined organic layers were dried over Na_2_SO_4_ and concentrated in vacuo. The product was purified by column chromatography on silica gel in 100% petroleum ether to afford the target compound **16** as colorless liquid (385 mg, 93%).

^1^H NMR (300 MHz, CDCl_3_), δ: 7.69 (dd, *J* = 7.2, 1.7 Hz, 1H), 7.45 (d, *J* = 5.5 Hz, 1H), 7.38–7.18 (m, 3H).

^13^C NMR (75 MHz, CDCl_3_), δ: 141.07, 139.13, 127.98, 127.43, 125.43, 124.63, 123.91, 122.07.

*(7-Chlorobenzo[b]thiophen-2-yl)boronic acid* (**17**), *n*-Butyllithium (2.5M solution in hexane, 2.5 mL, 6.25 mmol) was added dropwise to a 7-chlorobenzothiophene **16** (530 mg, 3.14 mol) precooled to −78° in 14 mL of THF under argon atmosphere. The resulting mixture was heated to −30 °C, kept at this temperature for 10 min and cooled down again to −78 °C, and then trimethylborate was added dropwise to the solution. The solution was allowed to warm up to 0 °C and was poured into the saturated NH_4_Cl solution (30 mL). The resulting mixture was partitioned with Et_2_O (3 × 20 mL). The ether solution was collected and evaporated to dryness. The remainder was dissolved in 2 M NaOH solution (20 mL) and stirred for 20 min. The obtained solution was washed with CH_2_Cl_2_. The aqueous layer was treated with 10% HCl (aq.) to reach pH = 1. The precipitate was filtered, washed with water, and dried in high vacuo to afford the target compound **17** as a yellow powder (400 mg, 60%).

^1^H NMR (300 MHz, DMSO-d6), δ: 8.04 (s, 1H), 7.90 (d, *J* = 7.7 Hz, 1H), 7.50 (d, *J* = 7.7 Hz, 1H), 7.41 (t, *J* = 7.7 Hz, 1H).

*Propargyl bromide*, The solution of phosphorus tribromide (53.0 g, 196.2 mmol) in CH_2_Cl_2_ (100 mL) was added dropwise to the solution of propargyl alcohol (10.0 mL, 173.40 mmol) in CH_2_Cl_2_ (100 mL) at 0 °C. The reaction mixture was stirred overnight, cooled in an ice bath, and quenched dropwise with water (125 mL). The organic layer was separated, washed with saturated NaHCO_3_ (aq.) and NaCl (aq.) solutions and dried over Na_2_SO_4_. The solvent was carefully evaporated, and the remainder was distilled at atmospheric pressure to afford the target compound as colorless liquid (BP = 85 °C, 17.4 g, 84%).

^1^H NMR (300 MHz, CDCl_3_), δ: 3.88 (d, *J* = 2.6 Hz, 2H, CH_2_), 2.53 (t, *J* = 2.6 Hz, 1H, CH).

*6-Bromo-1-(prop-2-yn-1-yl)-1H-indole* (**18**), 6-Bromoindol (1.00 g, 5.10 mmol) was added to the suspension of NaH (60% in oil, 265 mg, 6.60 mmol, pre-washed with petroleum ether) in absolute DMSO (20 mL) and was kept stirring until the gas release stopped. Propargyl bromide (1.24 mL, 6.60 mmol) was added dropwise to the resulting solution under thermal control with a water bath for heat dissipation. The mixture was stirred for 3 h. Water (40 mL) was added dropwise to the resulting mixture and pre-cooled in an ice bath. The mixture was extracted with EtOAc (3 × 40 mL). The combined organic layers were washed with H_2_O, with brine, dried over Na_2_SO_4_ and evaporated in vacuo. The crude product was purified by silica gel column chromatography (eluent: petroleum ether−AcOEt, 40:1) to afford the target compound **18** as a yellow oil (760 mg, 64%).

^1^H NMR (300 MHz, CDCl_3_), δ: 7.58–7.52 (m, 1H), 7.47 (d, *J* = 8.4 Hz, 1H), 7.27–7.10 (m, 2H), 6.49 (dd, *J* = 3.2, 0.9 Hz, 1H), 4.80 (d, *J* = 2.6 Hz, 2H, CH_2_), 2.41 (t, *J* = 2.6 Hz, 1H, CH).

^13^C NMR (75 MHz, CDCl_3_), δ: 136.61, 127.93, 127.78, 123.23, 122.33, 115.58, 112.47, 102.37 (Ar), 74.02 (CH), 35.95 (CH_2_).

*Methyl 5-((6-bromo-1H-indol-1-yl)methyl)-1H-pyrazole-3-carboxylate* (**19**), Methyl diazoacetate (632 mg, 6.32 mmol) was added dropwise to the solution of 6-bromo-1-(prop-2-yn-1-yl)-1H-indole **18** (740 mg, 3.16 mol) in 15 mL of toluene. The reaction mixture was refluxed for 6 h. The solvent was evaporated. The crude product was purified by silica gel column chromatography (eluent: petroleum ether−AcOEt, 1:1) to afford the target compound **19** as a brown solid (760 mg, 64%).

^1^H NMR (300 MHz, CDCl_3_), δ: 7.54–7.51 (m, 1H), 7.49–7.41 (m, 1H), 7.19 (dd, *J* = 8.4, 1.7 Hz, 1H), 7.12 (d, *J* = 3.2 Hz, 1H), 6.53 (s, 1H), 6.49 (dd, *J* = 3.2, 0.9 Hz, 1H), 5.34 (s, 2H), 3.85 (s, 3H).

^13^C NMR (75 MHz, CDCl_3_), δ: 160.35 (COO), 136.91, 128.50, 127.62, 123.05, 122.27, 115.56, 112.52, 107.39, 102.40, 52.30 (CH_3_), 43.43 (CH_2_).

HRMS of C_14_H_12_^79^BrN_3_O_2_ 334.0186 and C_14_H_12_^81^BrN_3_O_2_, *m*/*z*: calcd. for [M + H]^+^ 336.0166; found 334.0179 and 336.0161.

*Methyl 5-((6-(7-chlorobenzo[b]thiophen-2-yl)-1H-indol-1-yl)methyl)- 1H-pyrazole-3-carboxylate* (**20**), The mixture of methyl 5-((6-bromo-1H-indol-1-yl)methyl)- 1H-pyrazole-3-carboxylate **19** (350 mg, 1.00 mmol), (7-chlorobenzo[b]thiophen-2-yl)boronic acid (256 mg, 1.20 mmol), sodium carbonate (213 mg, 2.00 mmol) and Pd(dppf)Cl_2_ (40 mg, 0.05 mmol) was dissolved in the mixture of dioxane (1 mL) and water (1 mL) and was stirred at 90 °C for 4 h. All volatiles were evaporated, and the residue was dissolved in water. The solution was extracted with CH_2_Cl_2_. The combined organic layers were washed with water, dried over Na_2_SO_4_ and concentrated in vacuo. The solid product **20** was used further without purification and characterization.

*5-((6-(7-Chlorobenzo[b]thiophen-2-yl)-1H-indol-1-yl)methyl)-1H-pyrazole-3-carboxylic acid* (**21**), The intermediate product from the previous step was dissolved in 10 mL of MeOH and treated with 1 mL of 30% KOH solution (aq.). The mixture was stirred overnight, treated with 10% HCl solution (aq.) and extracted with EtOAc. The combined organic layers were washed with brine, dried over Na_2_SO_4,_ and evaporated in vacuo. The crude product was purified by preparative HPLC (column—Supelco Ascentis C8, 5 μm, 250 mm × 10 mm, detection—220 nm, flow rate—2.5 mL/min, eluent—MeCN/H_2_O 25/75 containing 0.10 M of TFA, 10 mg of the crude mixture were separated per one run) to obtain the pure target compound **21** (65 mg, 16%).

^1^H NMR (300 MHz, DMSO-d6), δ: 8.03 (s, 1H), 7.91 (dd, *J* = 7.8, 1.1 Hz, 1H), 7.81 (s, 1H), 7.52–7.37 (m, 5H), 7.15 (dd, *J* = 8.4, 1.7 Hz, 1H), 6.54 (s, 1H), 6.48 (d, *J* = 3.1 Hz, 1H), 5.39 (s, 2H).

^13^C NMR (75 MHz, DMSO-d6), δ: 142.58, 141.74, 136.98, 133.92, 130.28, 127.71, 126.99, 126.24, 124.90, 123.57, 122.60, 122.48, 114.52, 113.35, 107.43, 101.84.

HRMS of C_21_H_14_ClN_3_O_2_S, *m*/*z*: calcd. for [M + H]^+^ 407.0495; found 407.0492.

## Data Availability

No data available.

## Supplementary Materials

The following supporting information can be downloaded at: https://www.mdpi.com/article/10.3390/molecules28083568/s1, NMR spectra are available online. S2: General technical data. S3: NMR spectral data for compounds.

## Sample Availability

Samples of the compounds are available from the authors.

## References

[1] Sun Q., Collins R., Huang S., Holmberg-Schiavone L., Anand G.S., Tan C.-H., Van-den-Berg S., Deng L.-W., Moore P.K., Karlberg T. (2009). Structural Basis for the Inhibition Mechanism of Human Cystathionine γ-Lyase, an Enzyme Responsible for the production of H_2_S. J. Biol. Chem..

[2] Shatalin K., Nuthanakanti A., Kaushik A., Shishov D., Peselis A., Shamovsky I., Pani B., Lechpammer M., Vasilyev N., Shatalina E. (2021). Inhibitors of bacterial H2S biogenesis targeting antibiotic resistance and tolerance. Science.

[3] Nudler E., Shatalin K., Shishov D., Fedichev P. (2019). Compounds and Methods for Treating Bacterial Infections.

[4] Taydakov I.V., Dutova T.Y., Sidorenko E.N., Krasnoselsky S.S. (2011). Convenient modification of the Leimgruber-Batcho indole synthesis: Reduction of 2-nitro-β-pyrrolidinostyrenes by the FeCl_3_–activated carbon–N_2_H_4_∙H_2_O system. Chem. Heterocycl. Compd..

[5] Sakamoto T., Kondo Y., Iwashita S., Yamanaka H. (1987). Condensed heteroaromatic ring systems. XII. Synthesis of indole derivatives from ethyl 2-bromocarbanilates. Chem. Pharm. Bull..

[6] Yang K., Zhou F., Kuang Z., Gao G., Driver T.G., Song Q. (2016). Diborane-Mediated Deoxygenation of o-Nitrostyrenes to Form Indoles. Org. Lett..

[7] Scott W.P., Johnson J.L., Gribble G.W. (2021). Concerning the preparation of 6-bromotryptamine. Tetrahedron.

[8] Fallon K.J., Wijeyasinghe N., Leventis A., Marin-Beloqui J.M., Toolan D.T.W., Al-Hashimi M., Bronstein H. (2021). Tyrian purple: An ancient natural dye for cross-conjugated n-type charge transport. J. Mater. Chem. C.

[9] Isaac M., Slassi M., Xin T., Arora J., O’Brien A., Edwards L., Tehim A. (2003). Design, synthesis and biological activity of novel dimethyl-{2-[6-substituted-indol-1-yl]-ethyl}-amine as potent, selective, and orally-Bioavailable 5-HT 1D agonists. Bioorg. Med. Chem. Lett..

[10] Clare D., Dobson B.C., Inglesby P.A., Aissa C. (2019). Chemospecific Cyclizations of α-Carbonyl Sulfoxonium Ylides on Aryls and Heteroaryls. Angew. Chem. Int. Ed..

[11] Wyeth C., Hu B., Jetter J.W. (2007). Substituted Indoles and Methods of Their Use.

[12] Petit S., Duroc Y., Larue V., Giglione C., Léon C., Soulama C., Artaud I. (2009). Structure-Activity Relationship Analysis of the Peptide Deformylase Inhibitor 5-Bromo-1H-indole-3-acetohydroxamic Acid. Chem. Med. Chem..

[13] Pattenden G., Palframan M. (2013). Indirect Support for a Stepwise Carbonium Ion Pathway Operating in (4+3)-Cycloaddition Reactions between Furanoxonium Ions and 1,3-Dienes. Synlett.

[14] Hashmi A.S.K., Wölfle M., Ata F., Hamzic M., Salathé R., Frey W. (2006). Gold Catalysis: Dihydroisobenzofurans and Isochromanes by the Intramolecular Furan/Alkyne Reaction. Adv. Synth. Catal..

[15] Madden J., Hallett D.J., Parkes A., Raoof A., Wang X. (2010). New Bradykinin B1 Antagonists.

[16] Kuruba B.K., Vasanthkumar S., Emmanuvel L. (2017). Rhodium-catalyzed synthesis of 2,3–Disubstituted N-methoxy pyrroles and furans via [3+2] cycloaddition between metal carbenoids and activated olefins. Tetrahedron.

[17] Brouwer W.G. (1999). Process for Synthesizing Substituted 2-Benzo[B] Thiophene Carboxylic Acids and Salts Thereof.

[18] Smith A.L., Brennan P.E., Demorin F.F., Liu G., Paras N.A., Retz D.M. (2006). Aaminopyrimidine Compounds and Methods of Use.

[19] Chen X., Coate H., Crew A.P., Dong H.-Q., Honda A., Mulvihill M.J., Tavares P.A., Wang J., Werner D.S., Mulvihill K.M. (2007). Fused Bicyclic mTOR Inhibitors. U.S. Patent.

[20] Shang H., Zou Z., Li L., Yuan F., Li X., Zhang T., Tian Y. (2021). Anthraquinone Natural Product Modified Derivative.

[21] Stephen A., Hashmi K., Krause N., Hashmi A.S.K. (2004). Synthesis of Allenes by Isomerization Reactions. Modern Allene Chemistry.

[22] Rajappa S., Gumaste V.K. (2013). Reactivity of Thiophenes, Oligothiophenes and Benzothiophenes. Advances in Heterocyclic Chemistry.

[23] Martin R., Buchwald S.L. (2008). Palladium-Catalyzed Suzuki−Miyaura Cross-Coupling Reactions Employing Dialkylbiaryl Phosphine Ligands. Acc. Chem. Res..

[24] Cheung E.Y., Pennington L.D., Bartberger M.D., Staples R.J. (2014). 2,2′-Bi[Benzo[b]Thiophene]: An Unexpected Isolation of the Benzo[b]Thiophene Dimer. Acta Crystallogr. C Struct. Chem..

[25] Ostrowska S., Rogalski S., Lorkowski J., Walkowiak J., Pietraszuk C. (2018). Efficient Homocoupling of Aryl- and Alkenylboronic Acids in the Presence of Low Loadings of [{Pd(μ–OH)Cl(IPr)}_2_]. Synlett.

[26] Jiang P., Che X., Liao Y., Huang H., Deng G.-J. (2016). Three-component 2-aryl substituted benzothiophene formation under transition-metal free conditions. RSC Adv..

