# Supplementary material for: Synthesis of the Indole-Based Inhibitors of Bacterial Cystathionine γ-Lyase NL1-NL3
Source: Molecules. 2023 Apr 19;28(8):3568. doi: 10.3390/molecules28083568 (PMC10147042; doi:10.3390/molecules28083568)

ELECTRONIC SUPPLEMENTARY MATERIAL

Synthesis of the Indole-Based Inhibitors of Bacterial Cystathionine γ-Lyase NL1-NL3

Konstantin V. Potapov [2,1,#], Roman A. Novikov [1,2,3,#,*], Maxim A. Novikov [2,#], Pavel N. Solyev [1,#,*], Yury V. Tomilov [2,*], Sergey N. Kochetkov [1], Alexander A. Makarov [1], Vladimir A. Mitkevich [1]

[1] Engelhardt Institute of Molecular Biology of the Russian Academy of Sciences, 32 Vavilov St., Moscow 119991, Russia; kospotapov@yandex.ru (K.V.P.); novikovfff@bk.ru (R.A.N.); solyev@gmail.com (P.N.S.), kochet@eimb.ru (S.N.K.); aamakarov@eimb.ru (A.A.M.); mitkevich@gmail.com (V.A.M.);

[2] Zelinsky Institute of Organic Chemistry of the Russian Academy of Sciences, 47 Leninsky avenue, 119991 Moscow, Russia; kospotapov@yandex.ru (K.V.P.); manovikov@ioc.ac.ru (M.A.N.); novikovfff@bk.ru (R.A.N.); tom@ioc.ac.ru (Y.V.T.);

[3] Biotechnology Department, Sirius University of Science and Technology, 1 Olympic Avenue, 354349, Sirius, Russia; novikovfff@bk.ru (R.A.N.);

* Correspondence: solyev@gmail.com (P.N.S.); Tel.: +7-499-135-60-65; novikovfff@bk.ru (R.A.N.); tom@ioc.ac.ru (Y.V.T.); Tel.: +7-499-135-63-90.

[#] K.V.P., M.A.N., R.A.N., P.N.S. contributed equally to this work.

Experimental section

1. General details S2

2. NMR spectral data for compounds S3

1.1. General experimental details

^{1}H, ^{13}C NMR spectra were recorded on a Bruker AVANCE II 300 MHz (300.1, 75.5 MHz and 282.4 MHz respectively) and a Bruker AMX III 400 MHz (400.1, 100.6 MHz and 376.5 MHz respectively) spectrometers in $CDCl_3$, containing 0.05% Me_4Si as the internal standard. Determination and verification of structures obtained compounds and assignments of ^{1}H and ^{13}C signals were made with the aid of 1D and 2D DEPT, COSY, HSQC and HMBC spectra.

4. NMR spectral data for compounds

^{1}H NMR (300 MHz) in $CDCl_3$

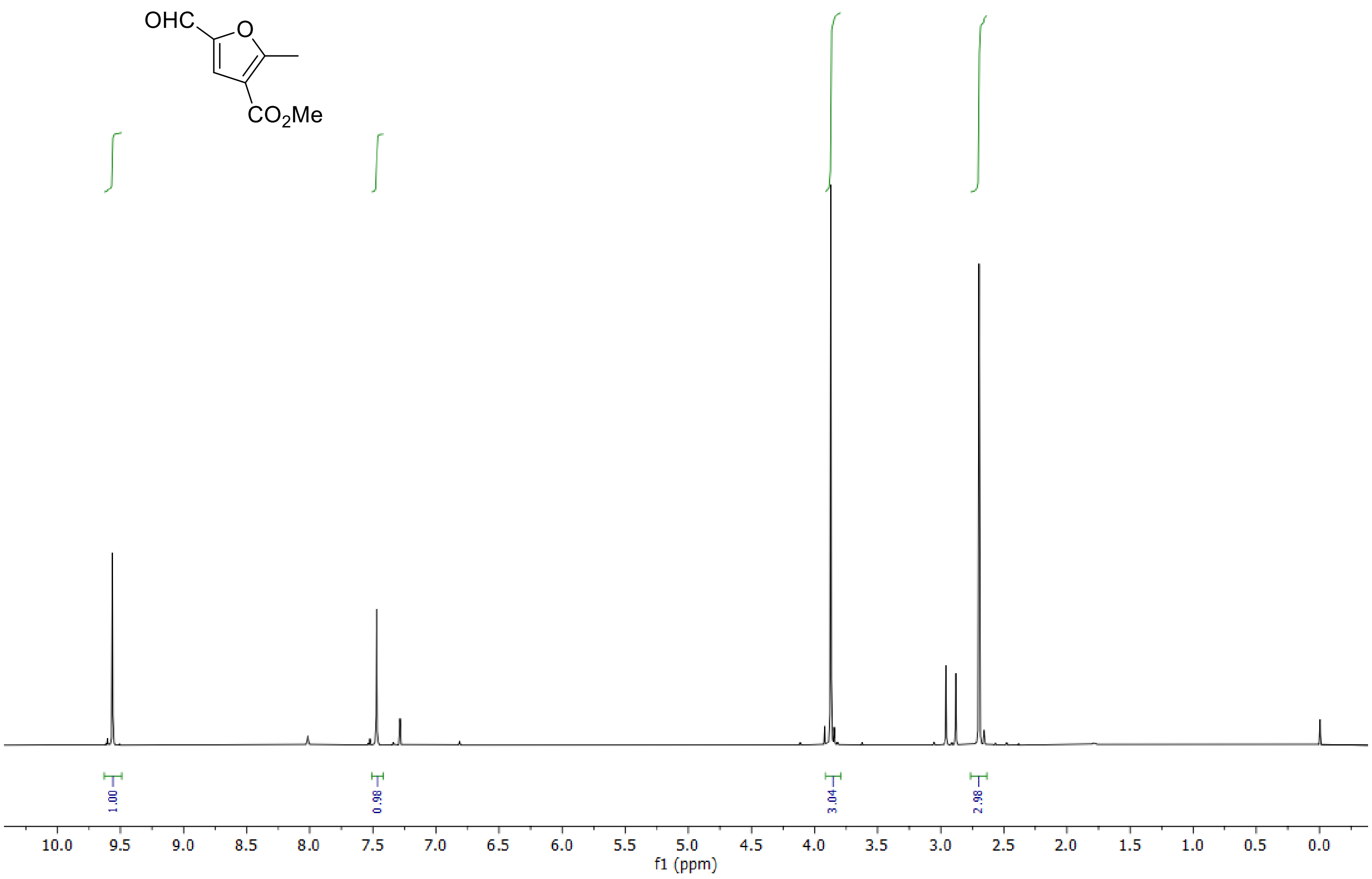

^{1}H NMR (300 MHz) in $CDCl_3$

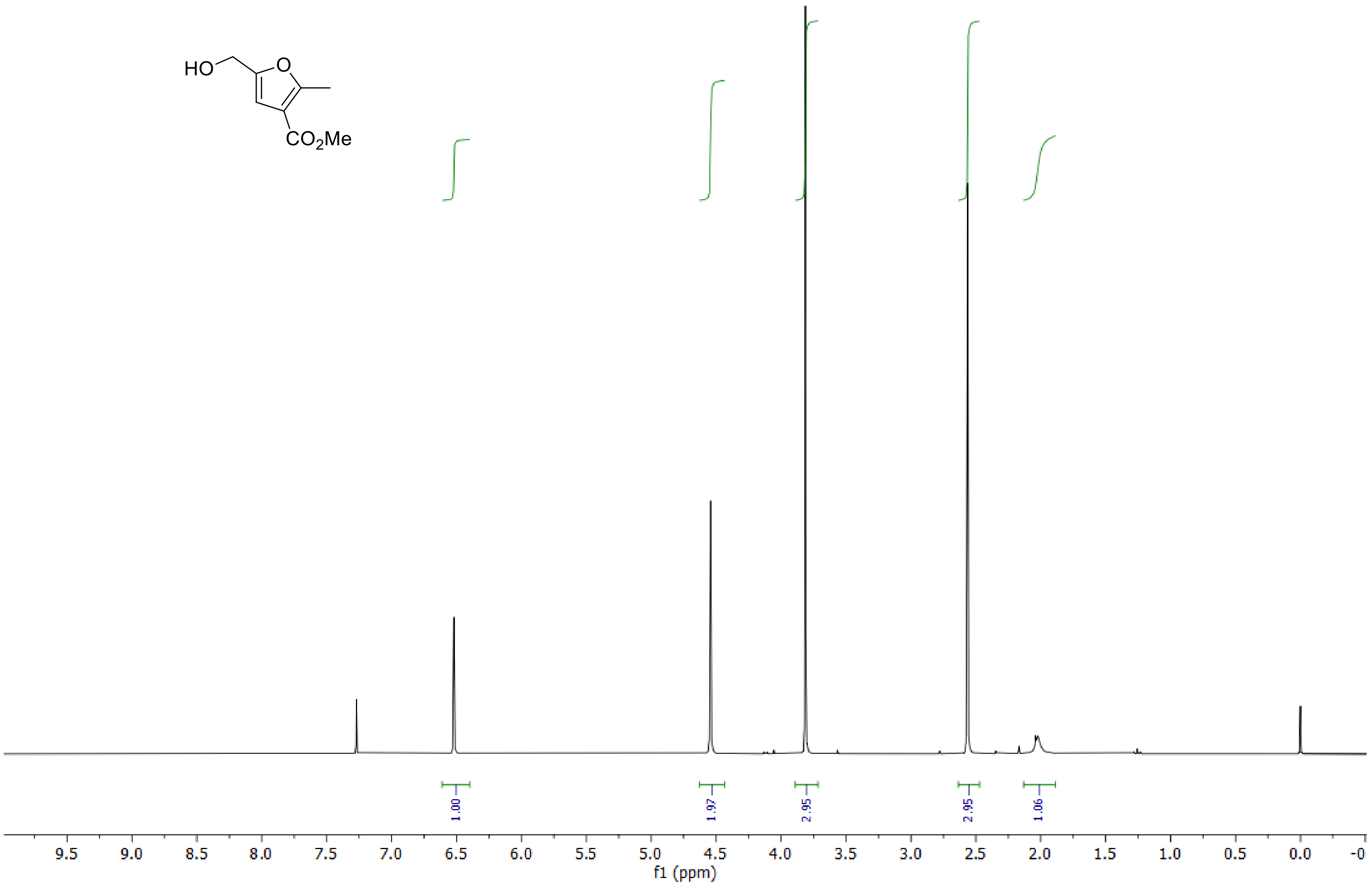

^{13}C NMR (76 MHz) in $CDCl_3$

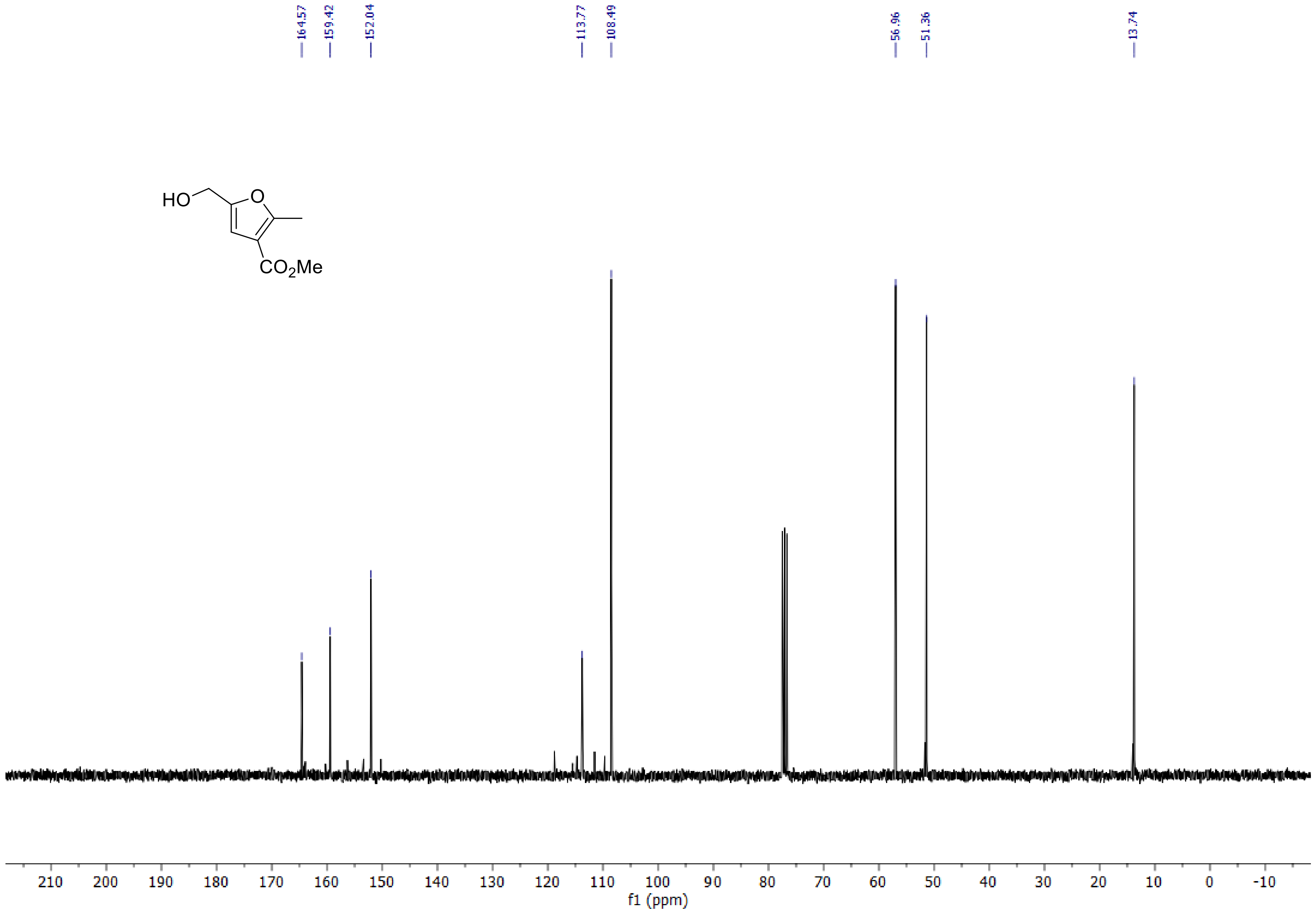

^{1}H NMR (300 MHz) in $CDCl_3$

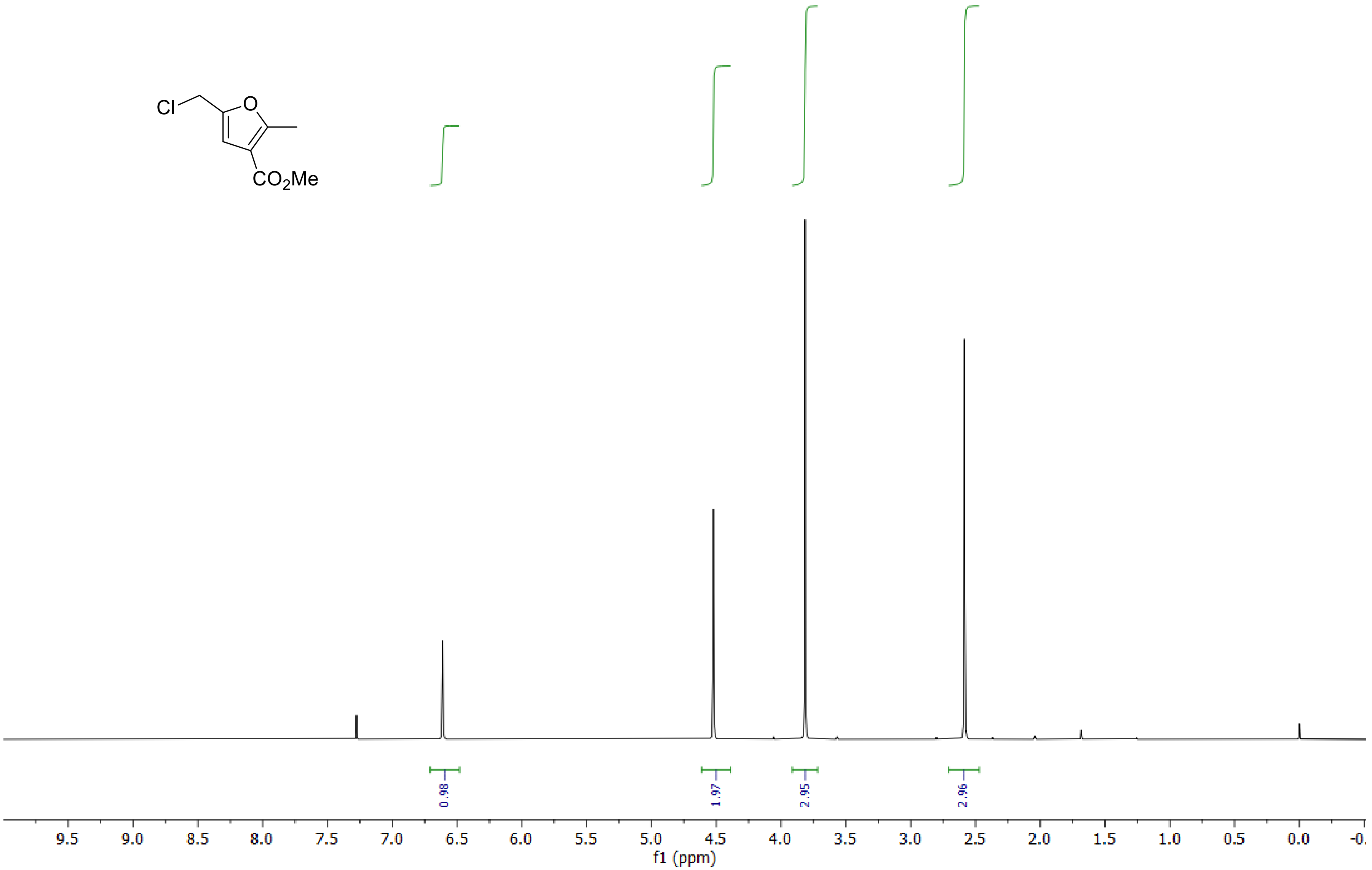

^{13}C NMR (76 MHz) in $CDCl_3$

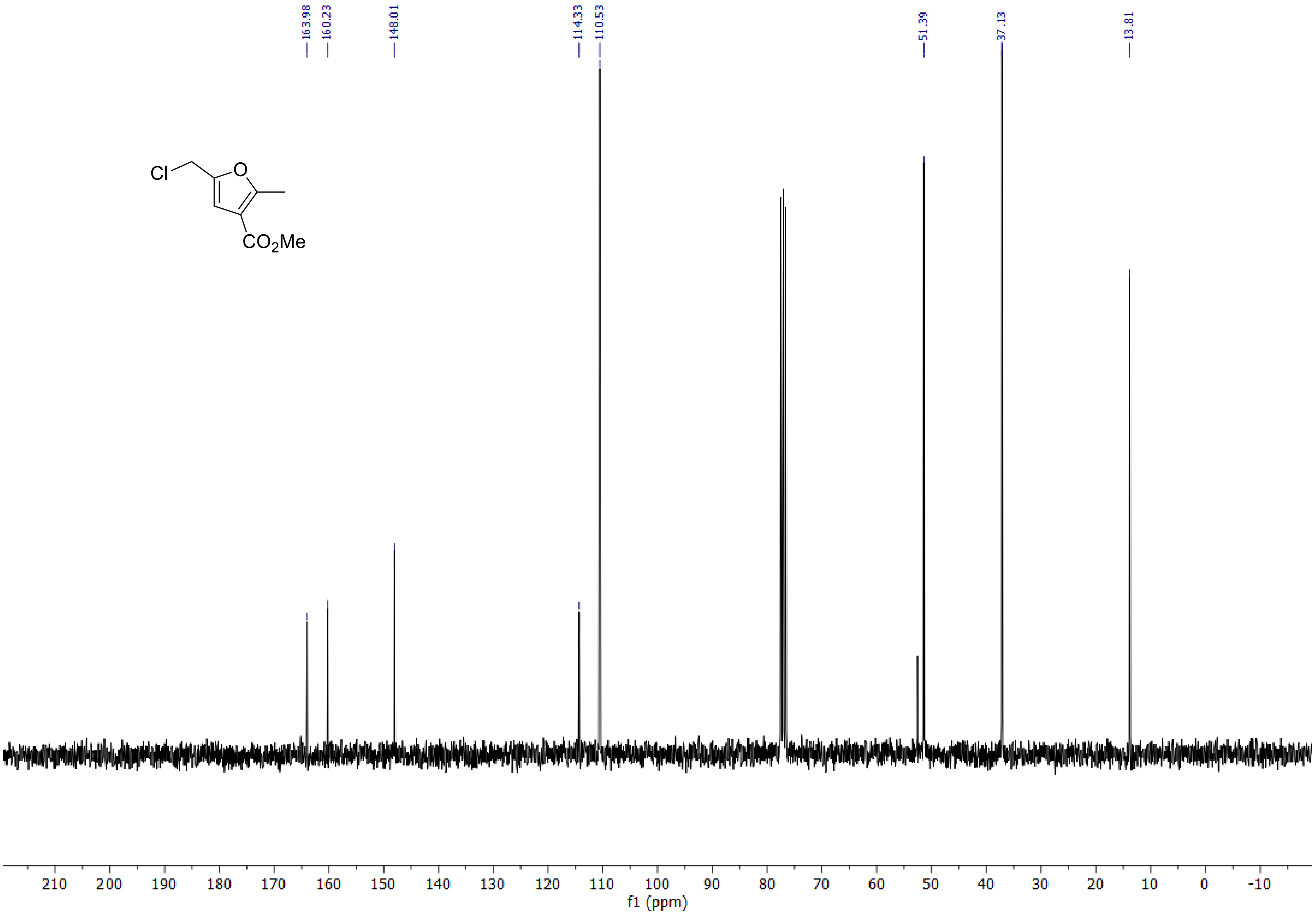

^{1}H NMR (300 MHz) in $CDCl_3$

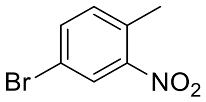

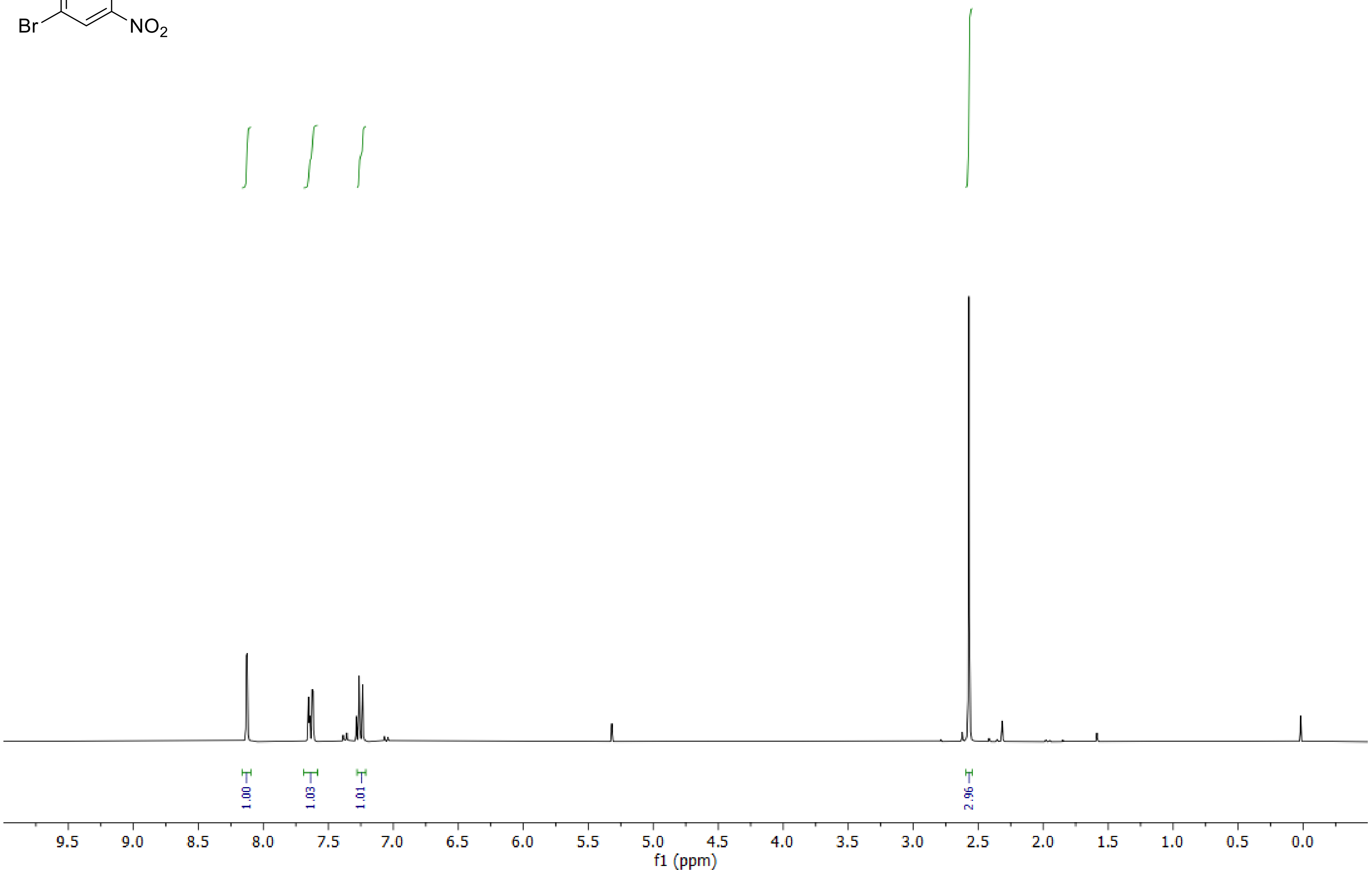

^{13}C NMR (76 MHz) in $CDCl_3$

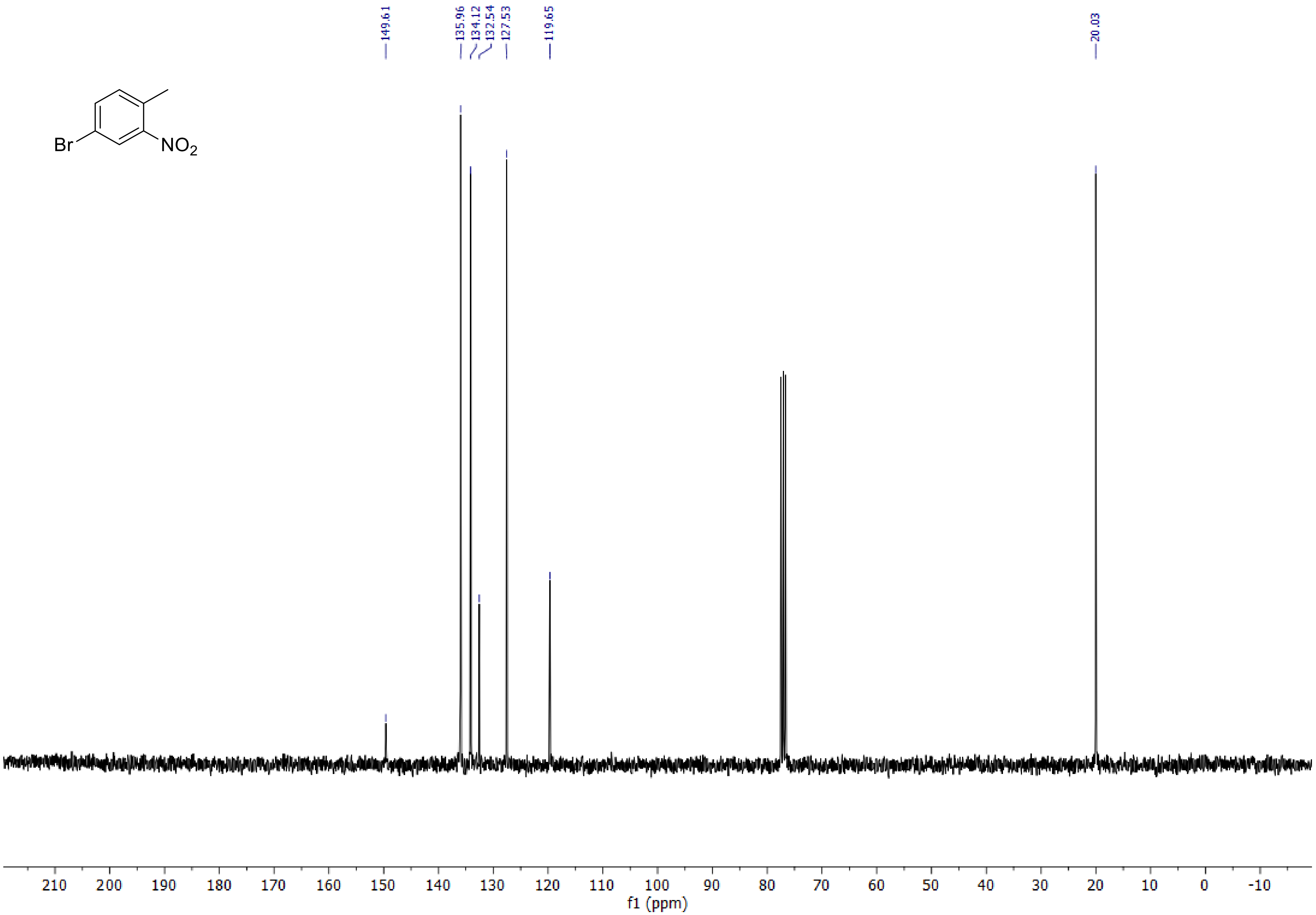

^{1}H NMR (300 MHz) in $CDCl_3$

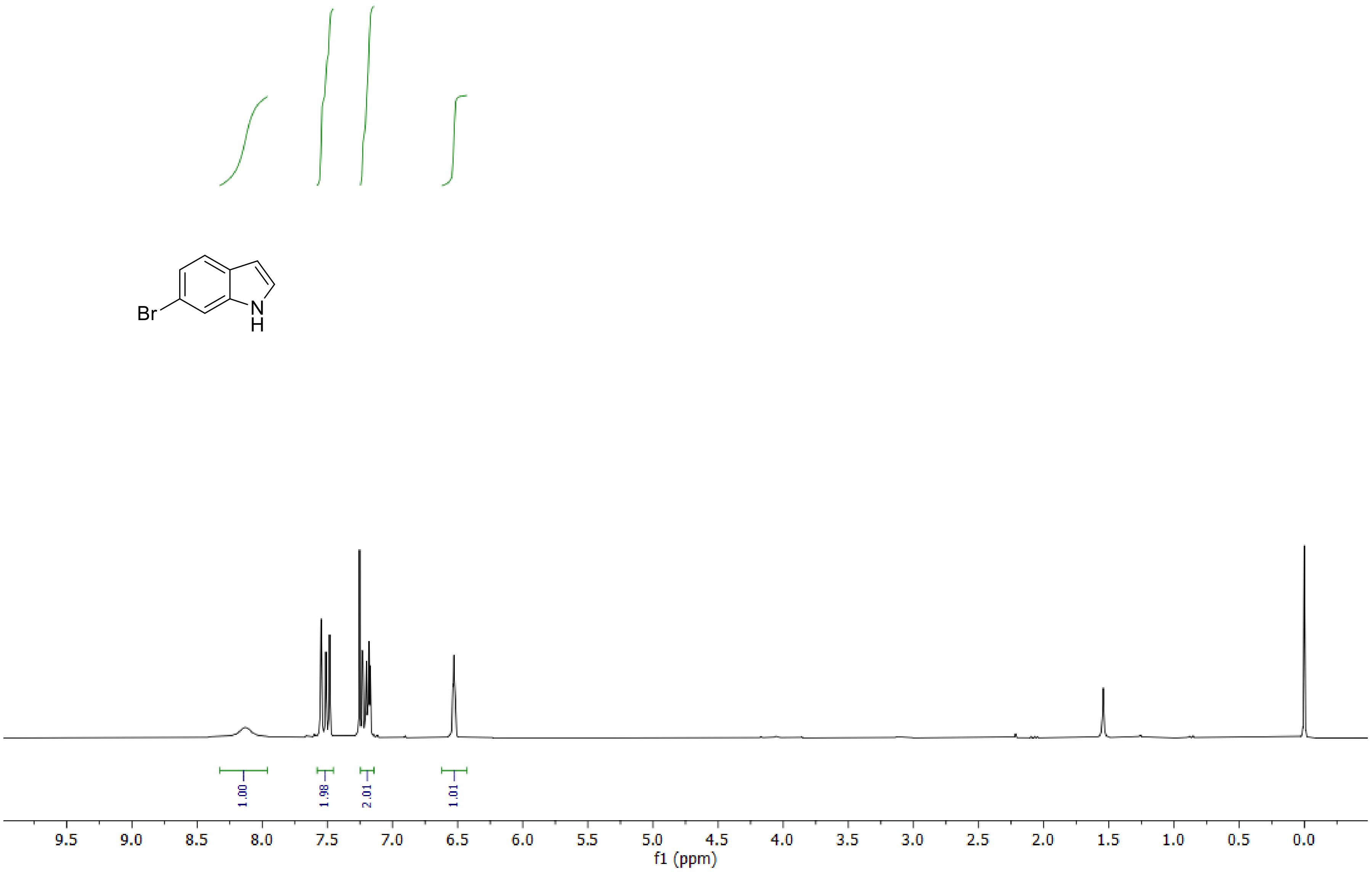

^{13}C NMR (76 MHz) in $CDCl_3$

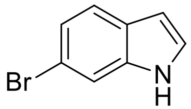

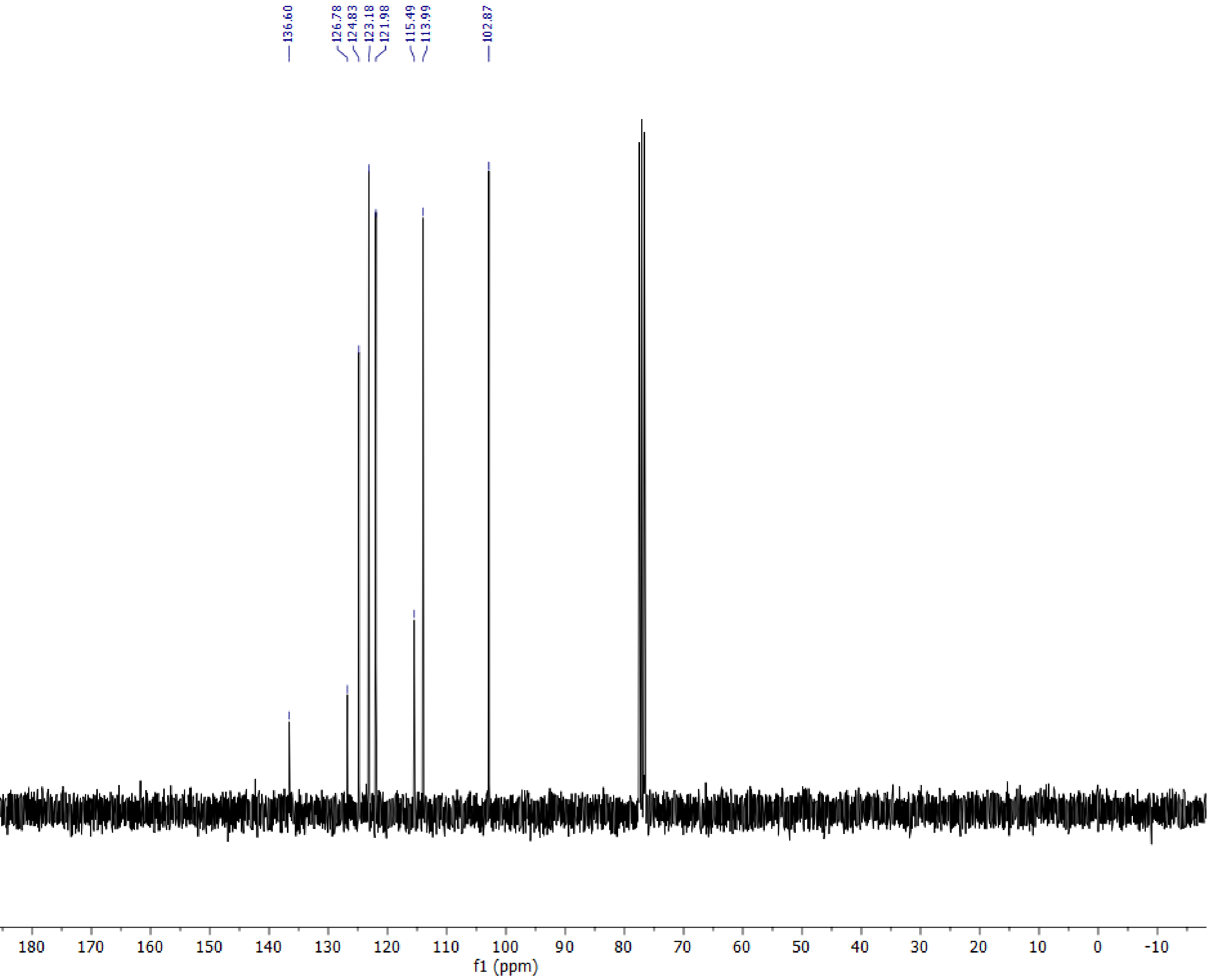

^{1}H NMR (300 MHz) in $CDCl_3$

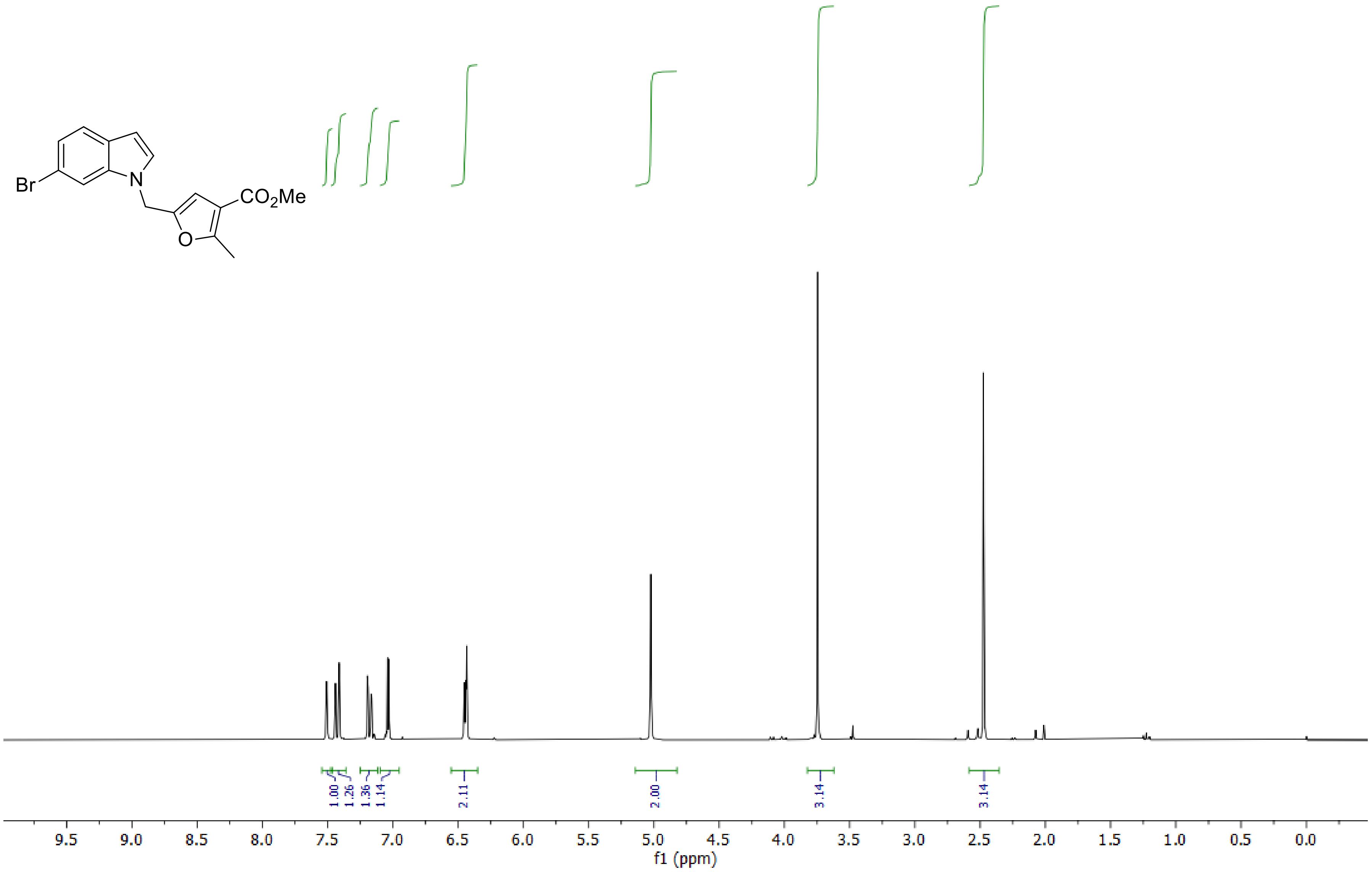

^{13}C NMR (76 MHz) in $CDCl_3$

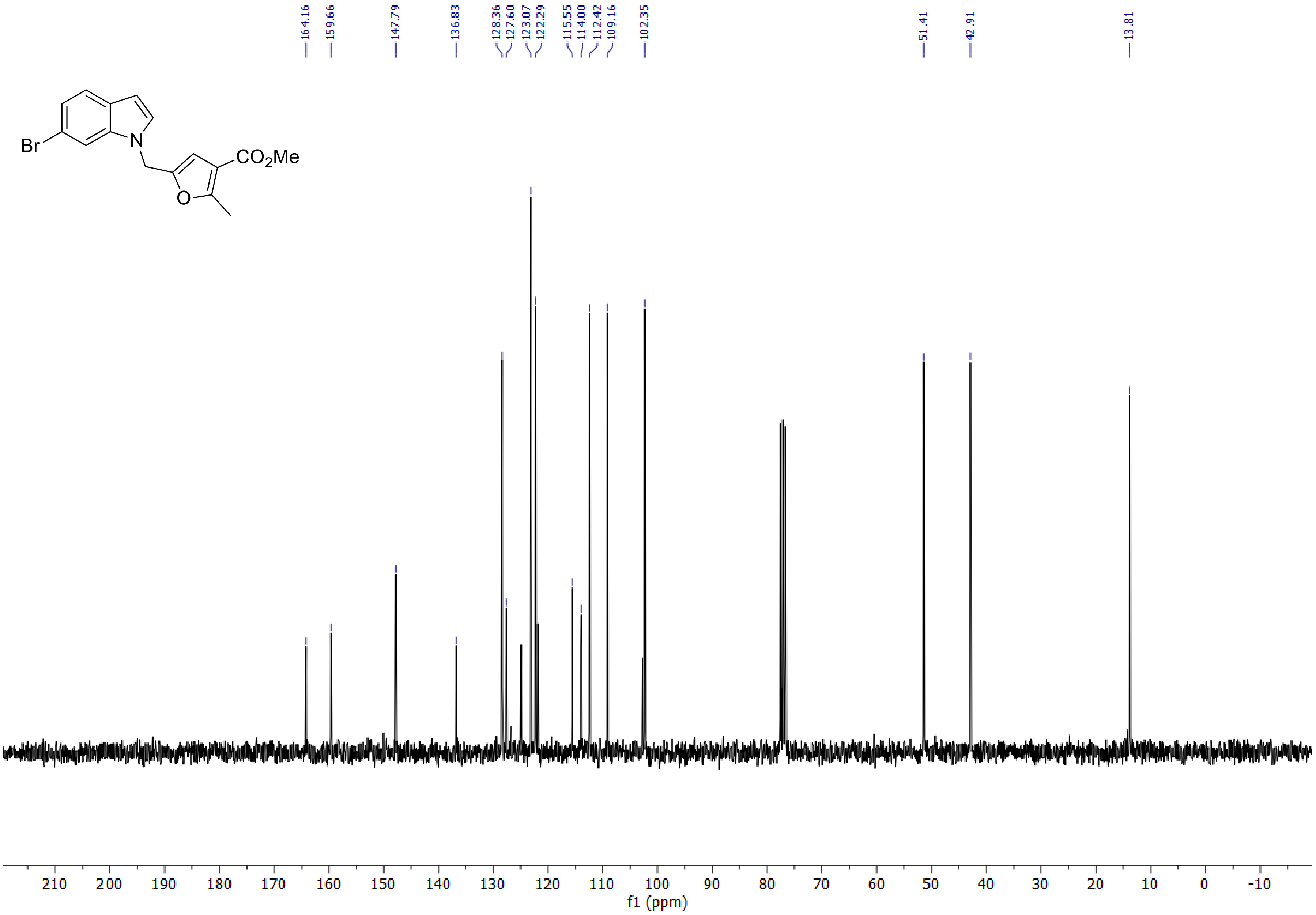

^{1}H NMR (400 MHz) in $CDCl_3$

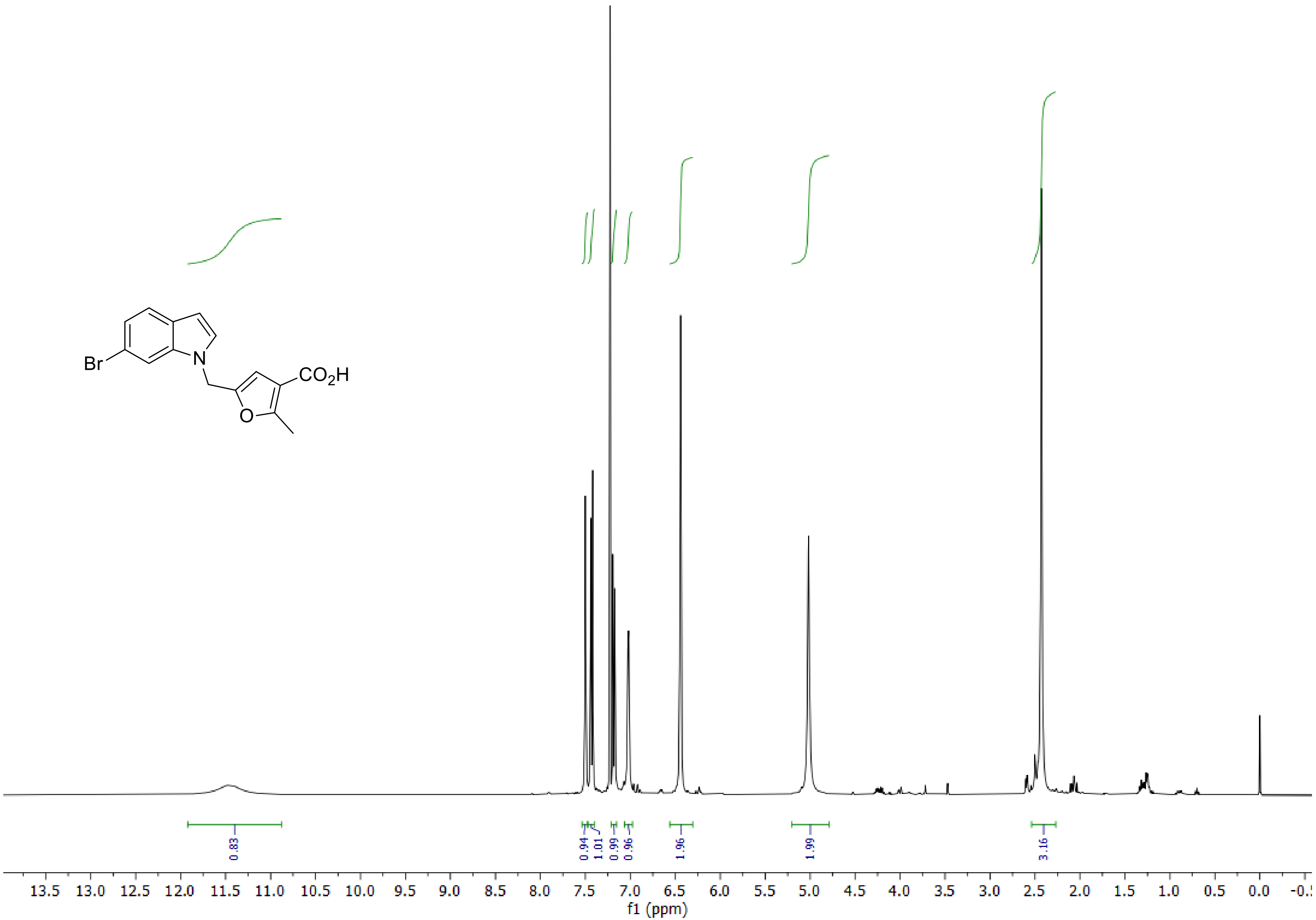

^{13}C NMR (101 MHz) in $CDCl_3$

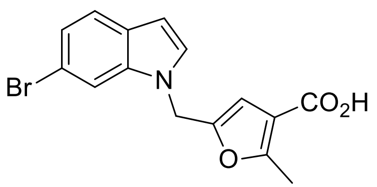

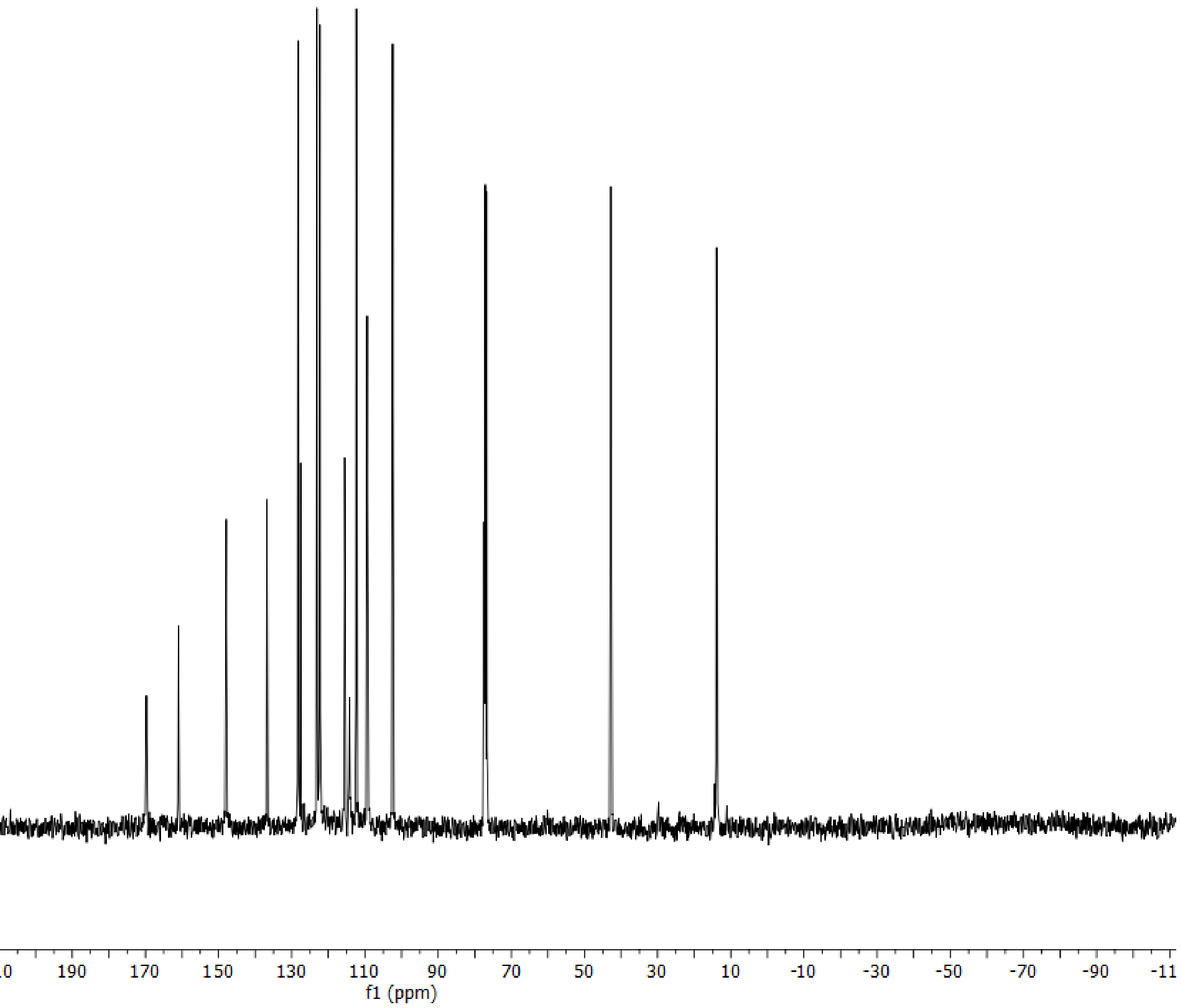

{13C}deptsp135 NMR (101 MHz) in $CDCl_3$

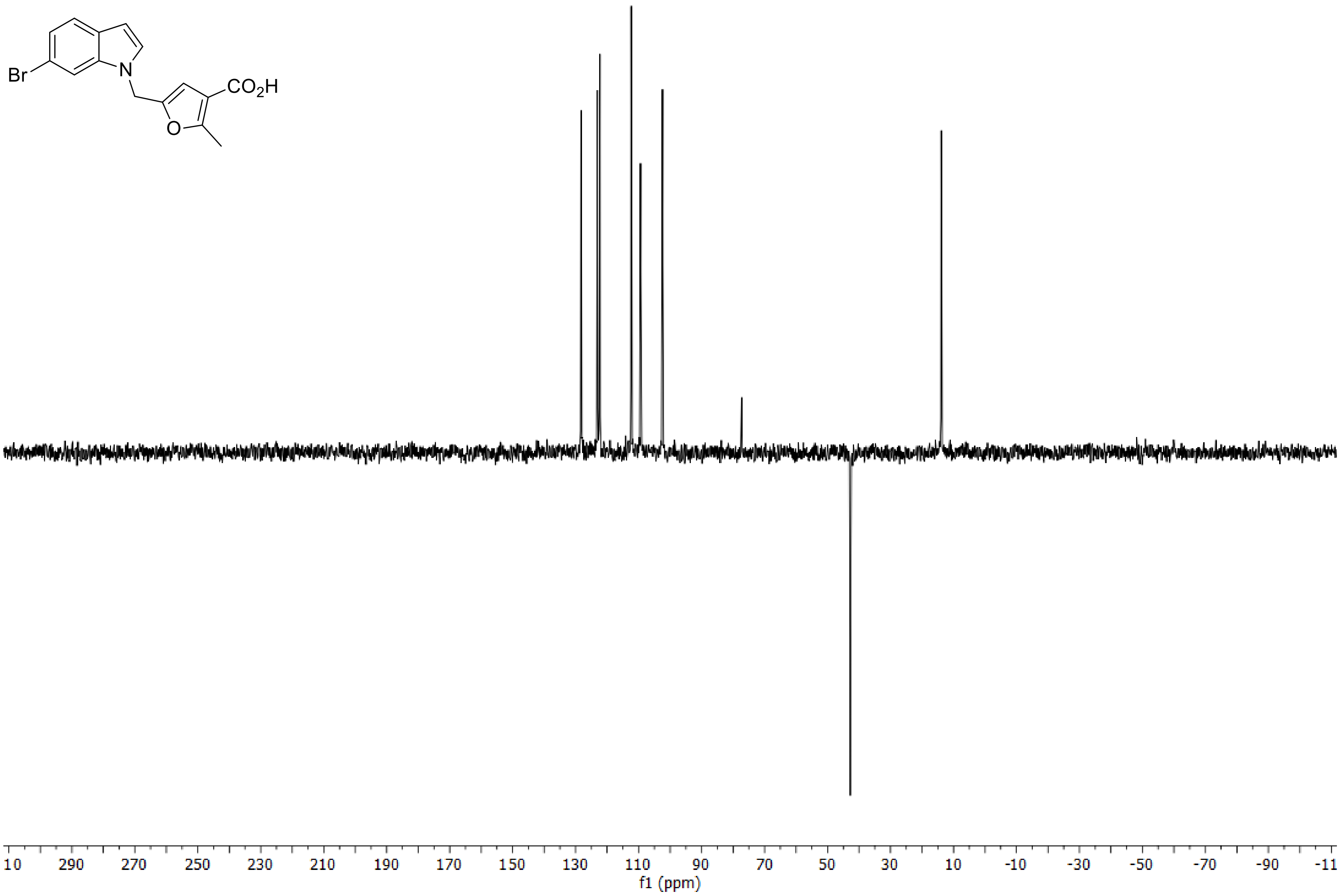

$^1H,^1H$-COSY (400 MHz) in $CDCl_3$

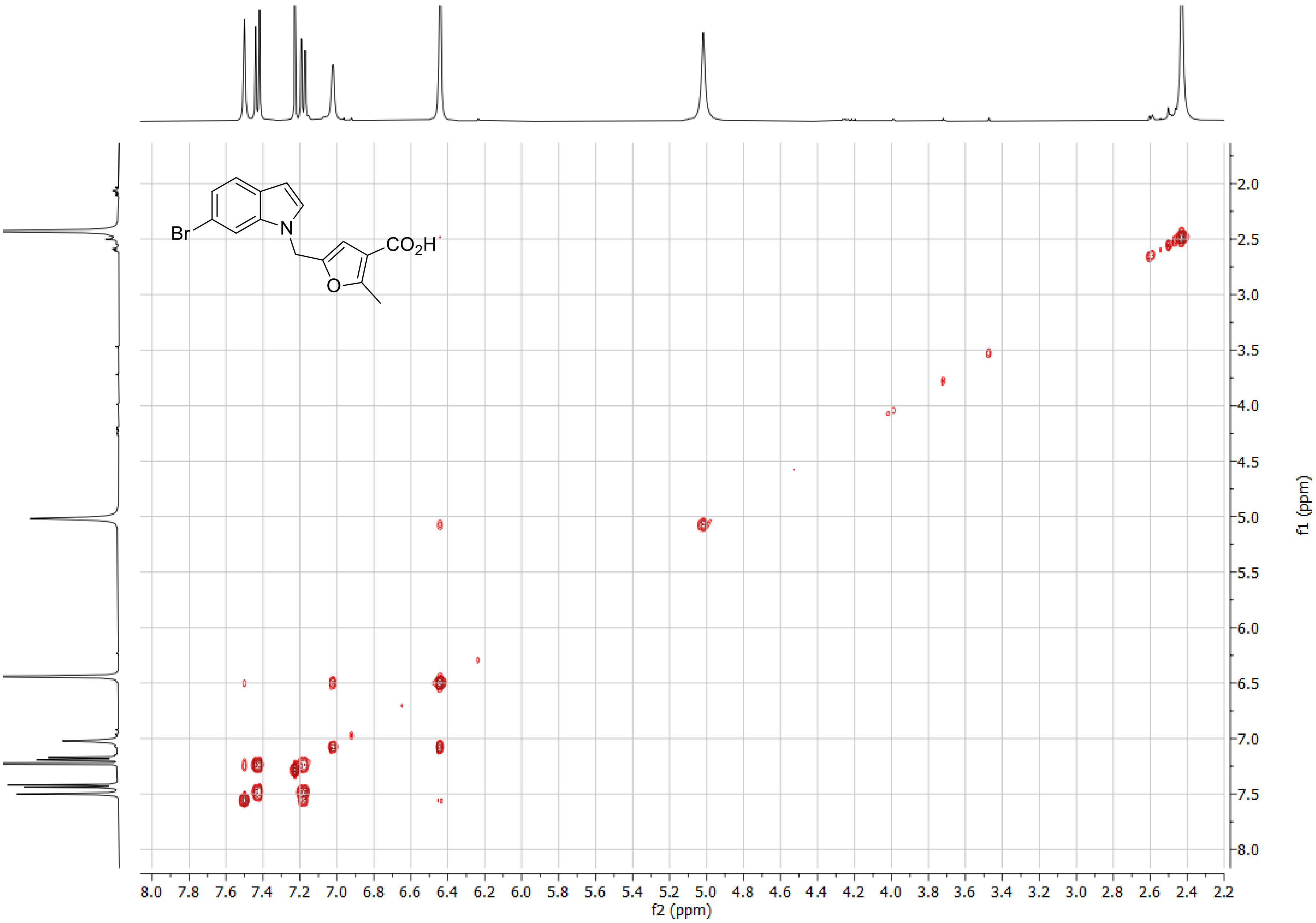

$^{1}H,^{13}C$-HSQC (400 & 101 MHz) in $CDCl_3$

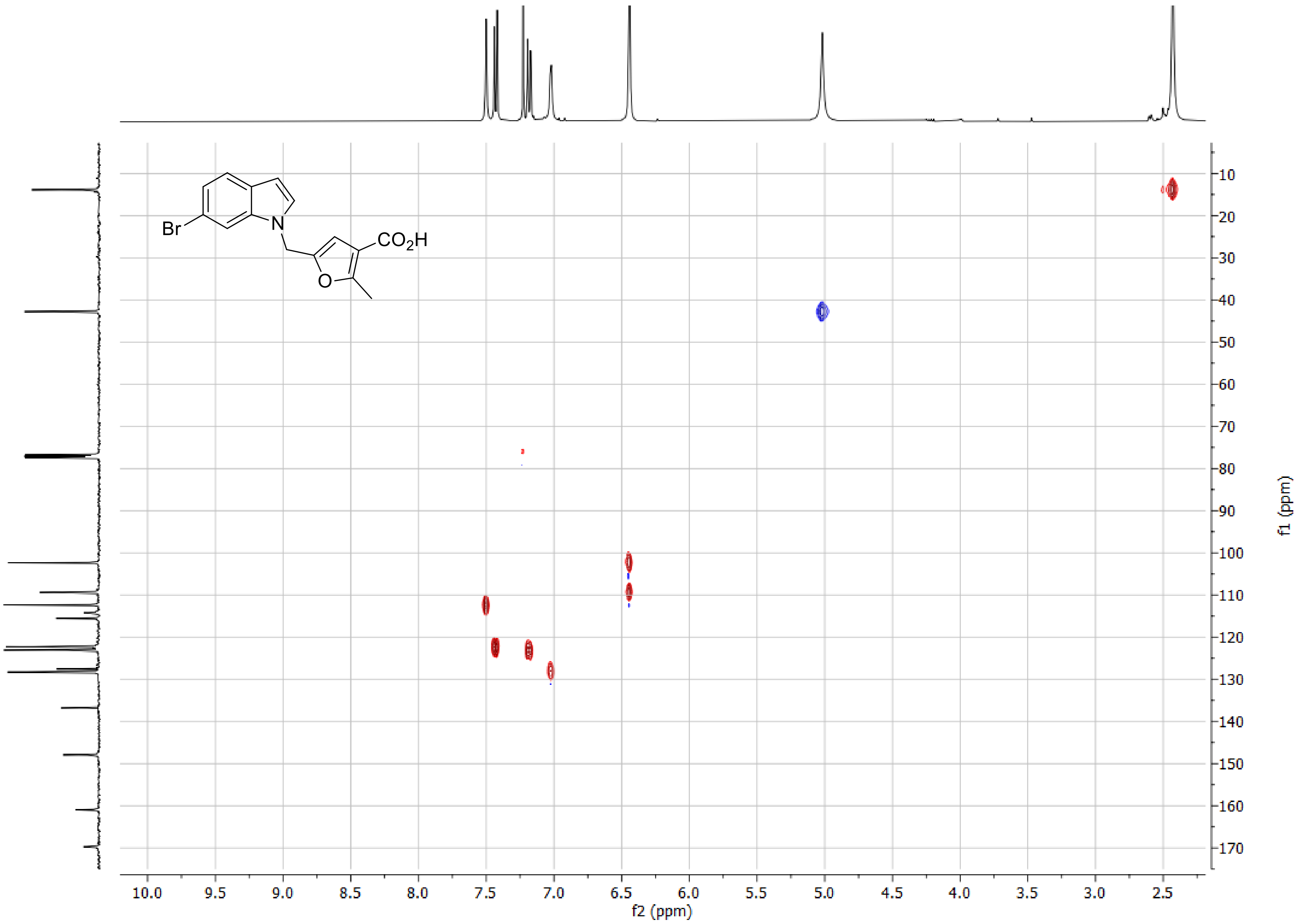

$^{1}H,^{13}C$-HMBC (400 & 101 MHz) in $CDCl_3$

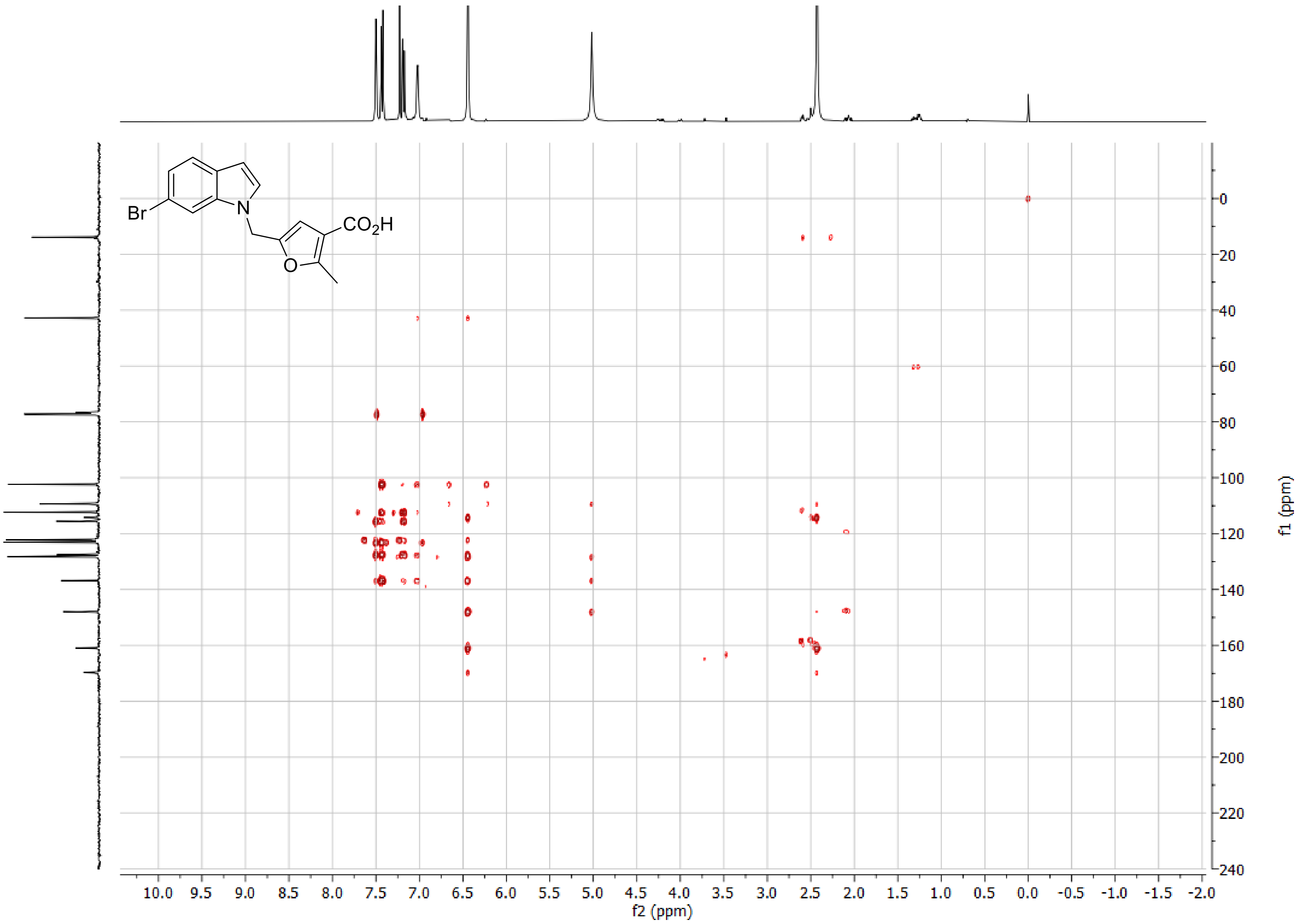

^{1}H NMR (300 MHz) in $CDCl_3$

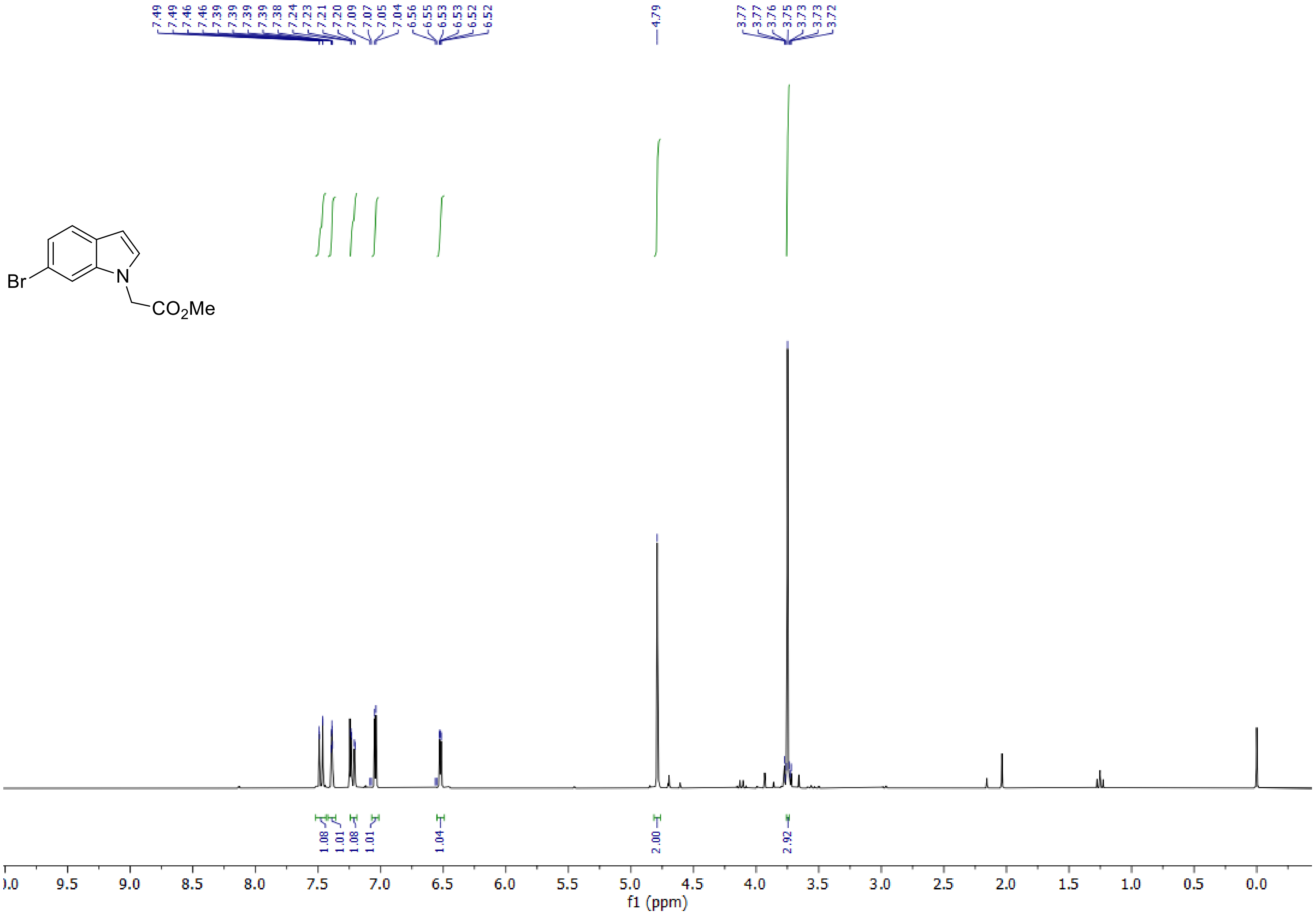

^{13}C NMR (75 MHz) in $CDCl_3$

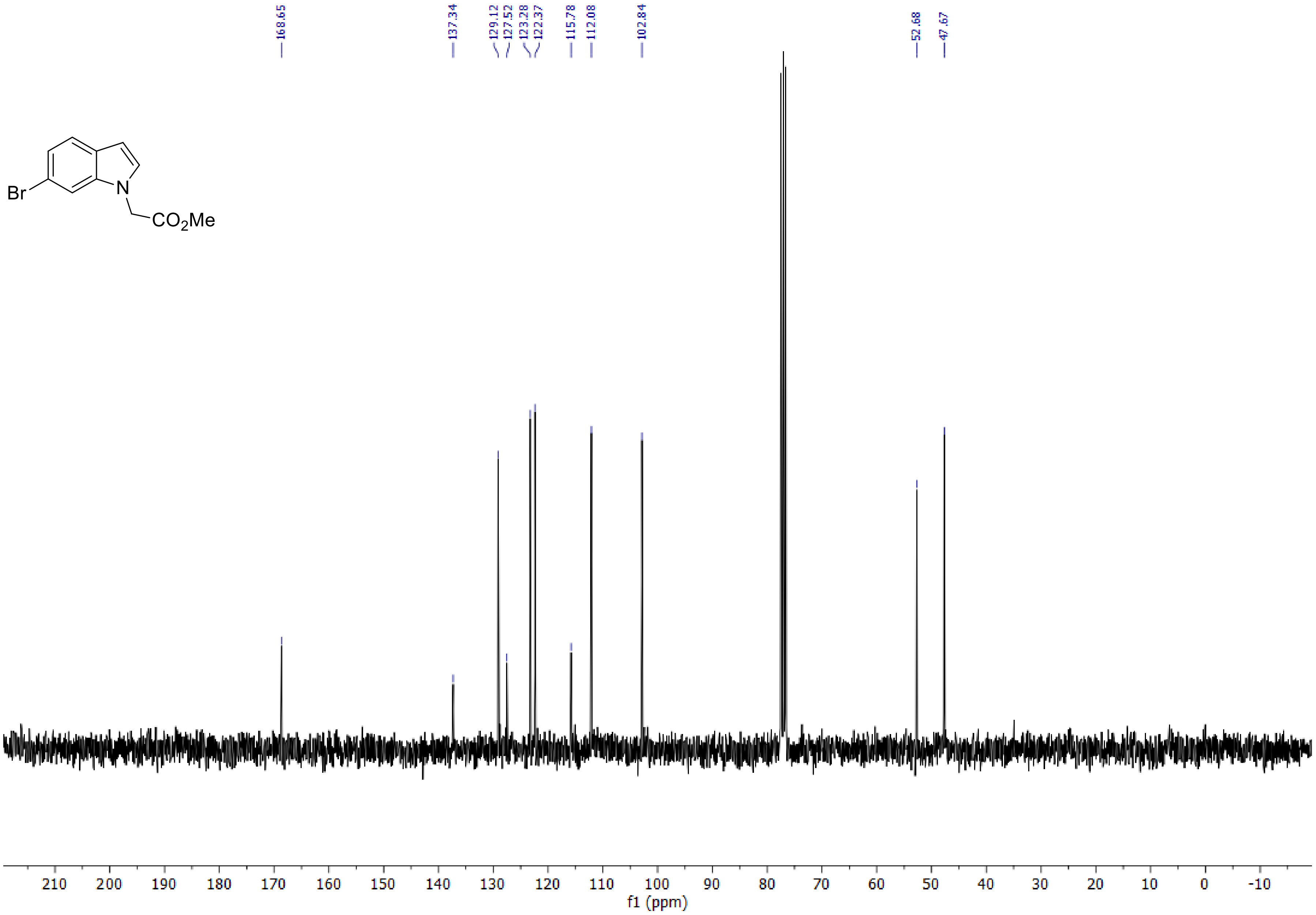

^1H NMR (300 MHz) in DMSO-d6

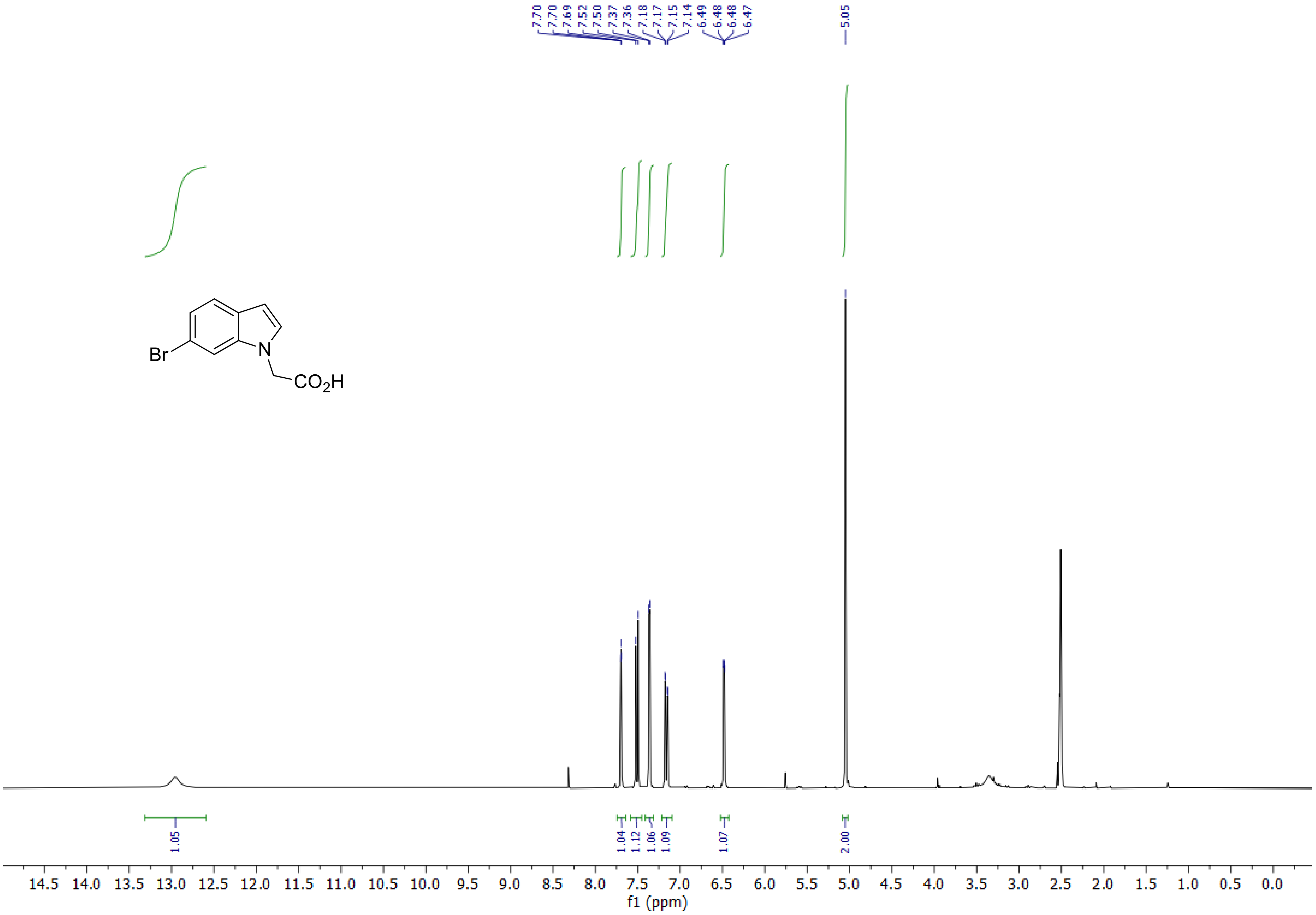

^{13}C NMR (75 MHz) in DMSO-d6

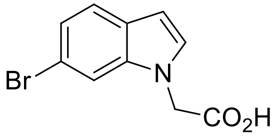

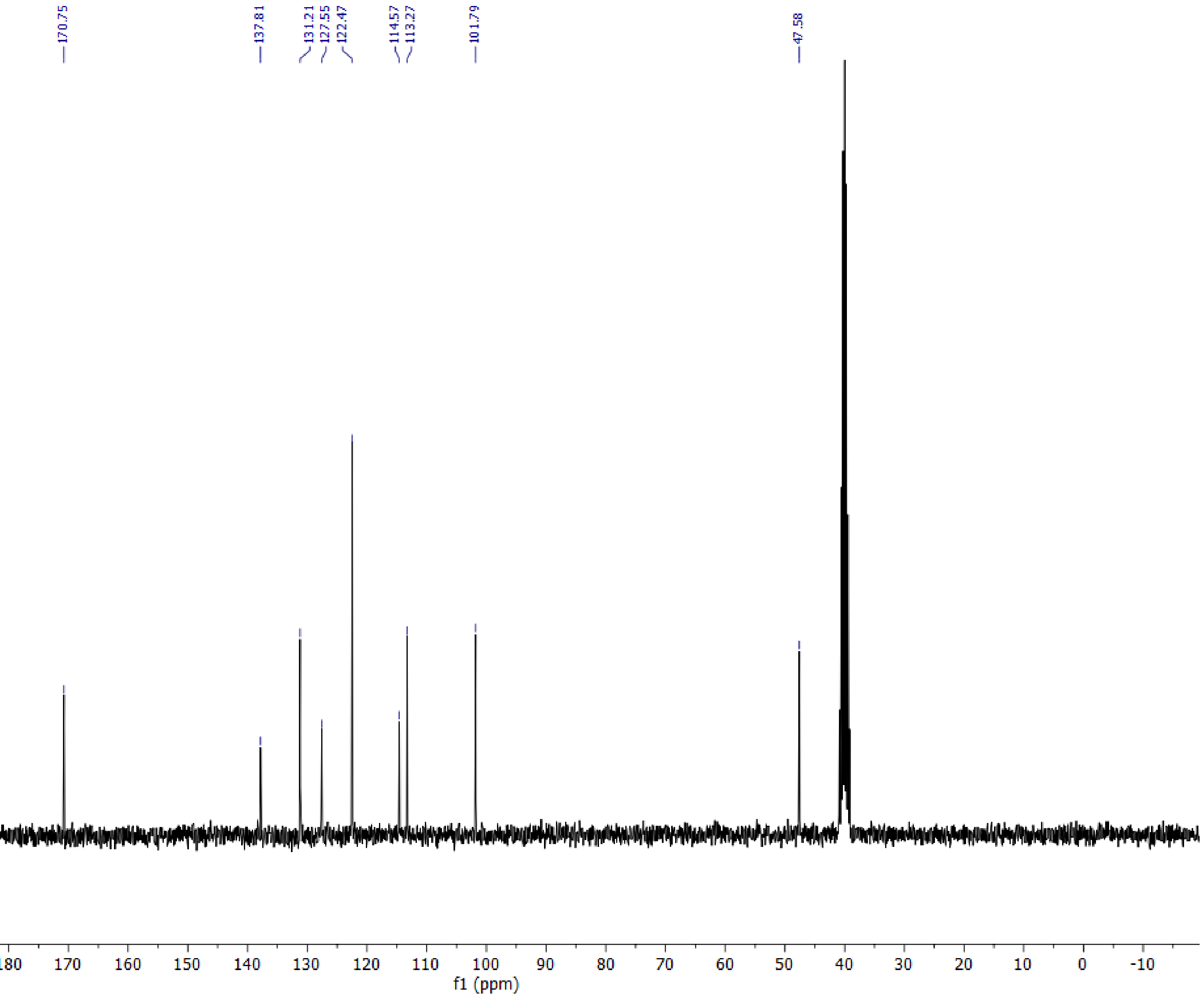

^1H NMR (300 MHz) in $CDCl_3$

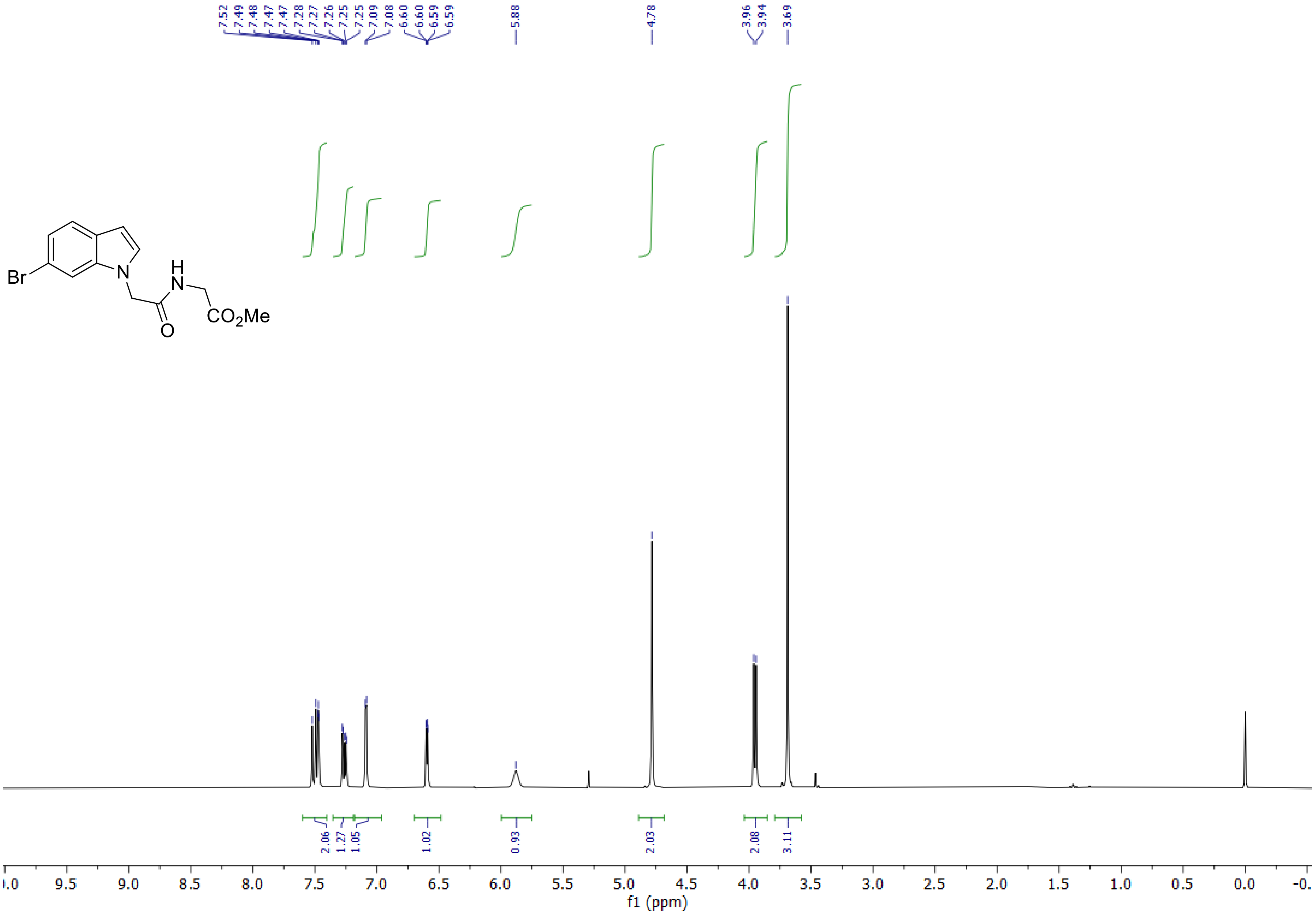

^{13}C NMR (75 MHz) in $CDCl_3$

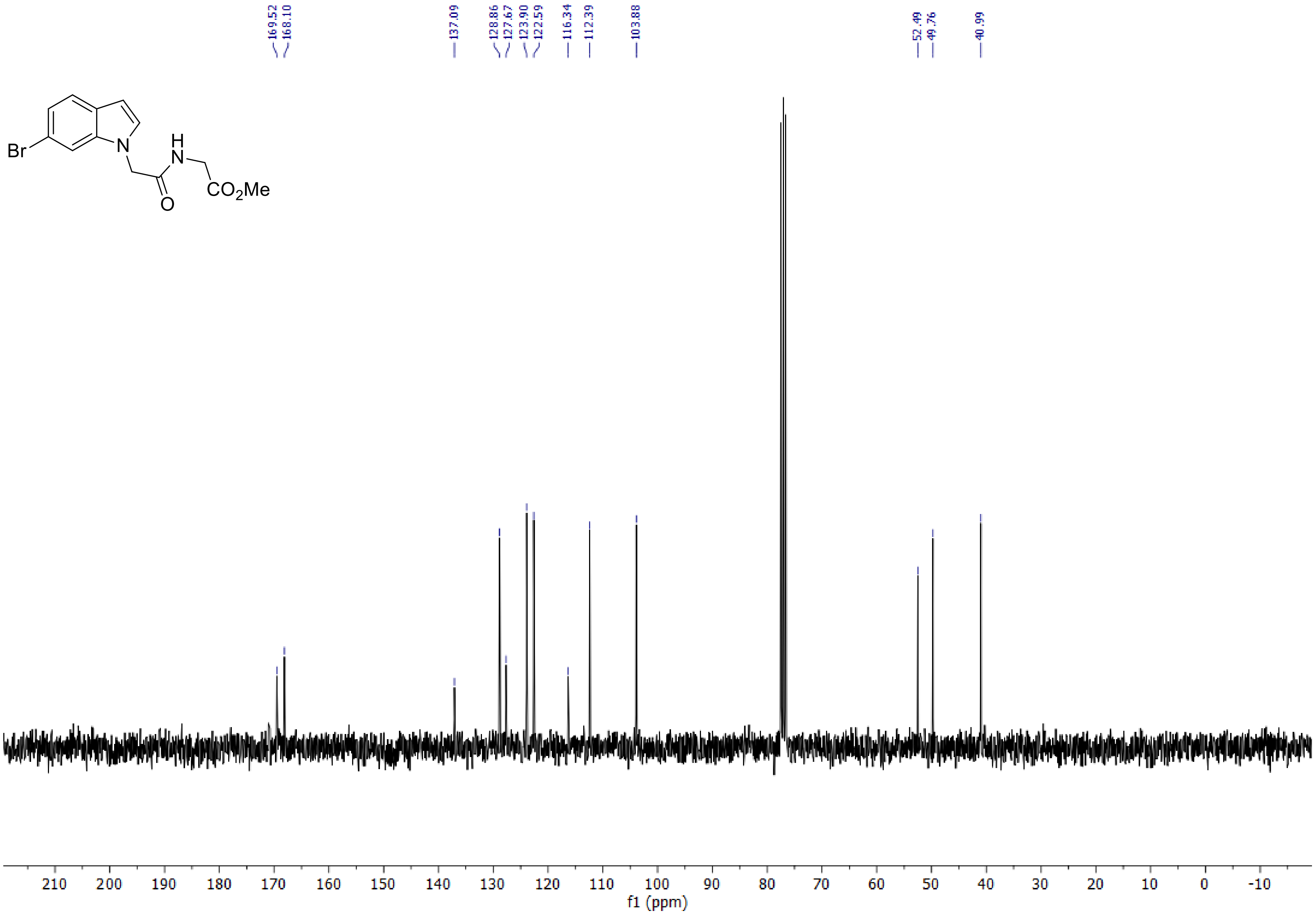

^{1}H NMR (300 MHz) in $CDCl_3$

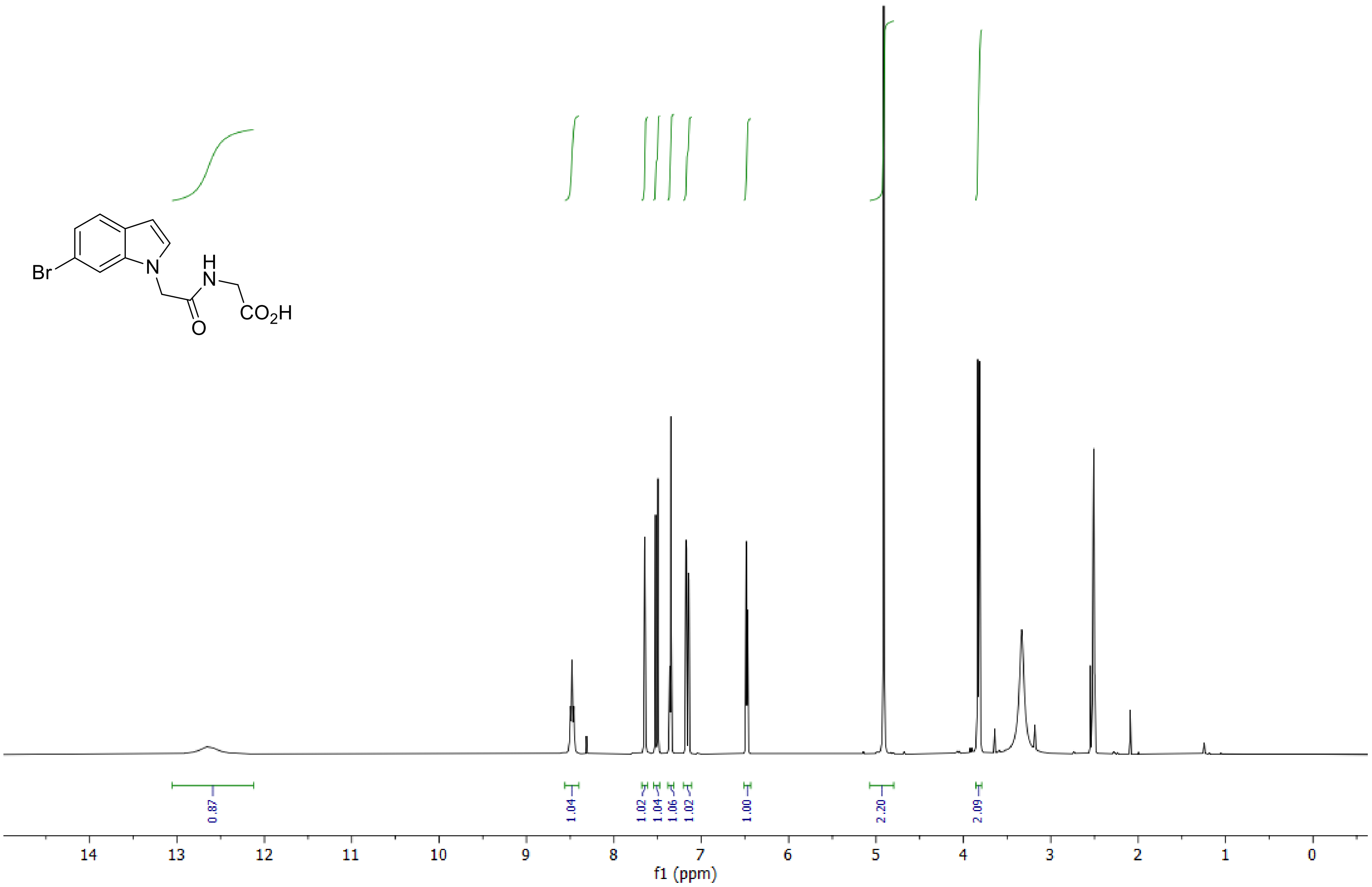

^{13}C NMR (75 MHz) in $CDCl_3$

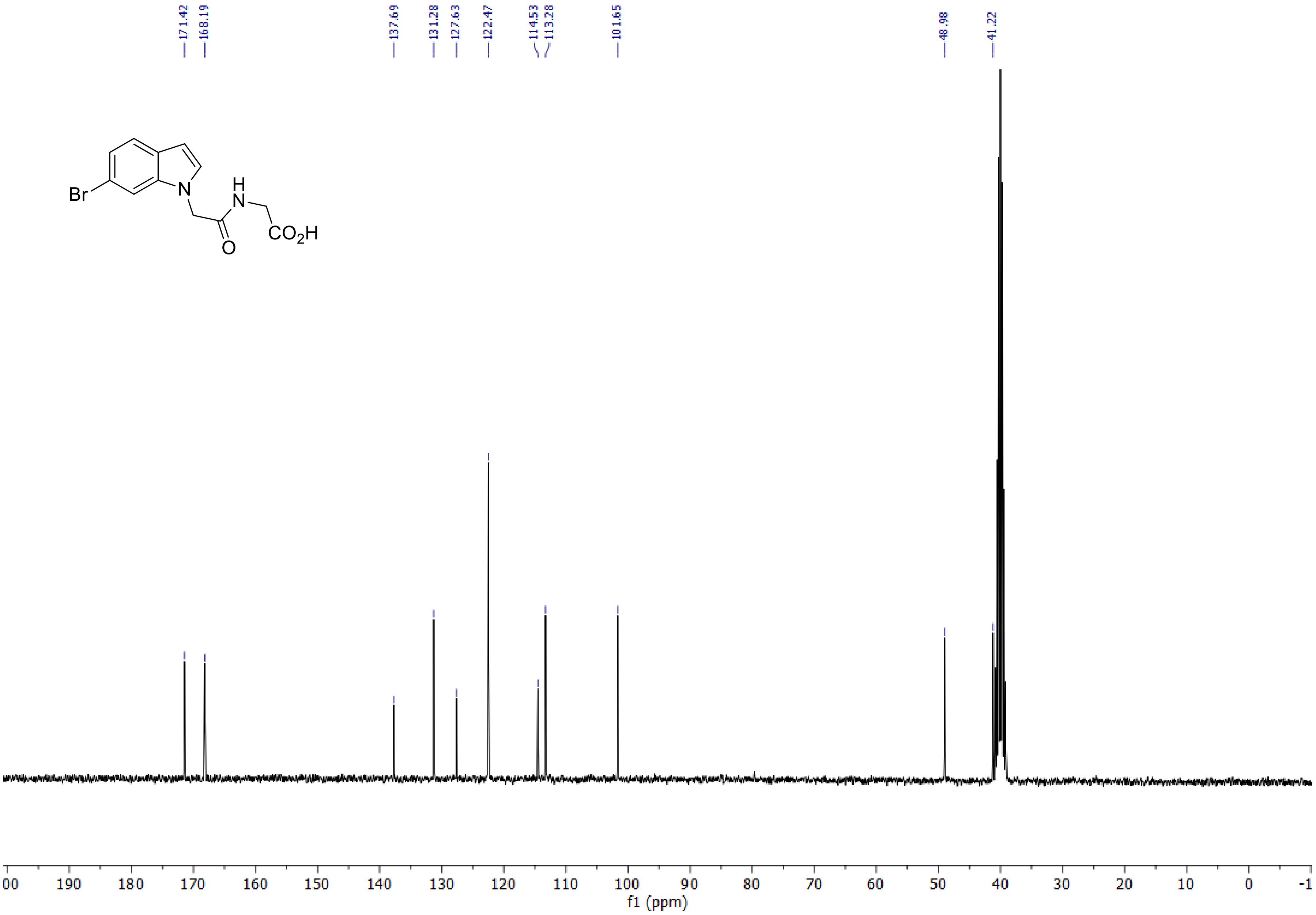

{13C}deptsp135 NMR (101 MHz) in $CDCl_3$

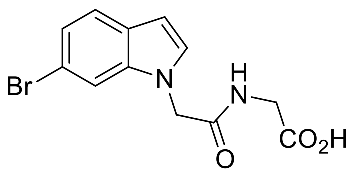

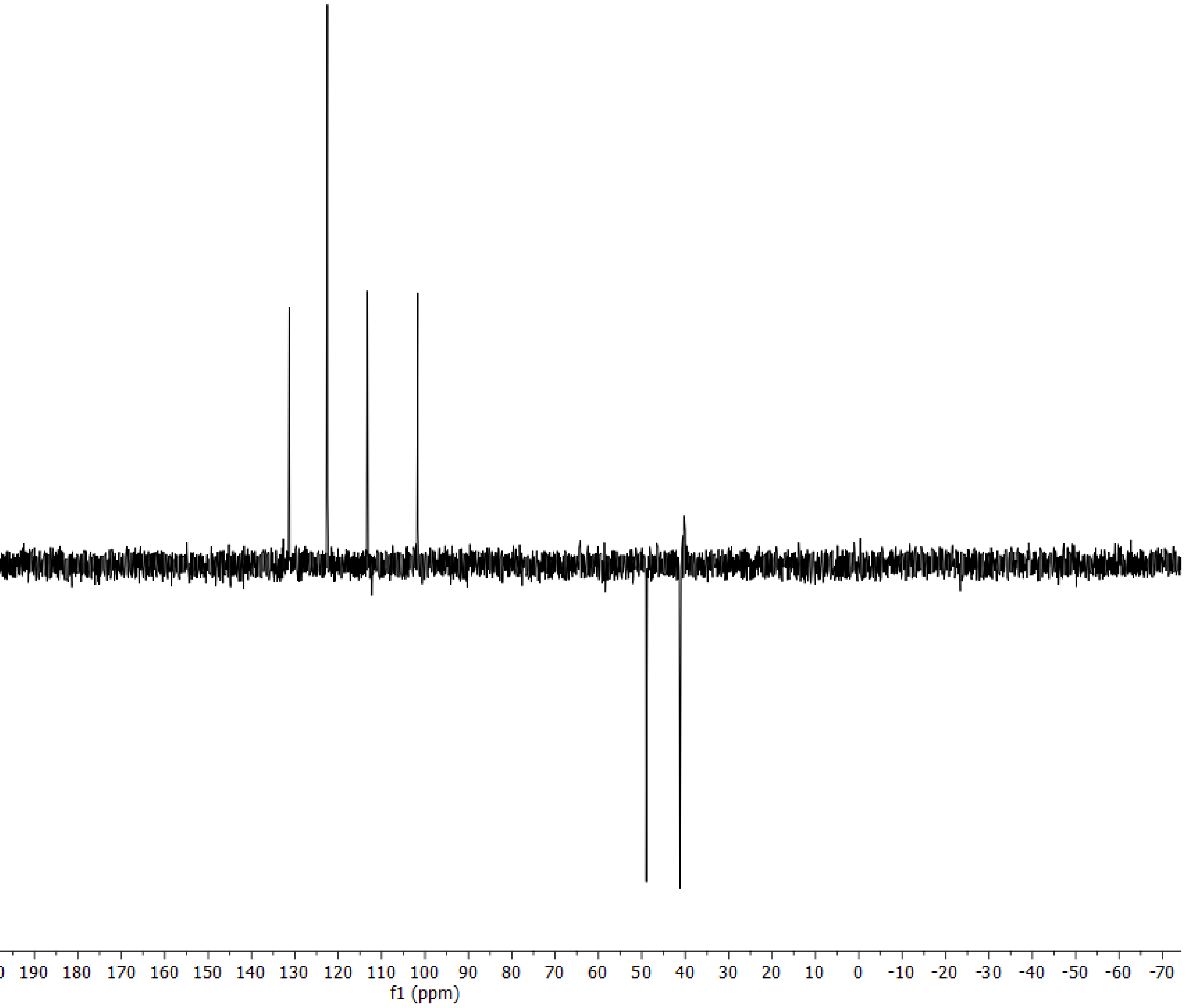

$^{1}H,^{1}H$-COSY (400 MHz) in $CDCl_3$

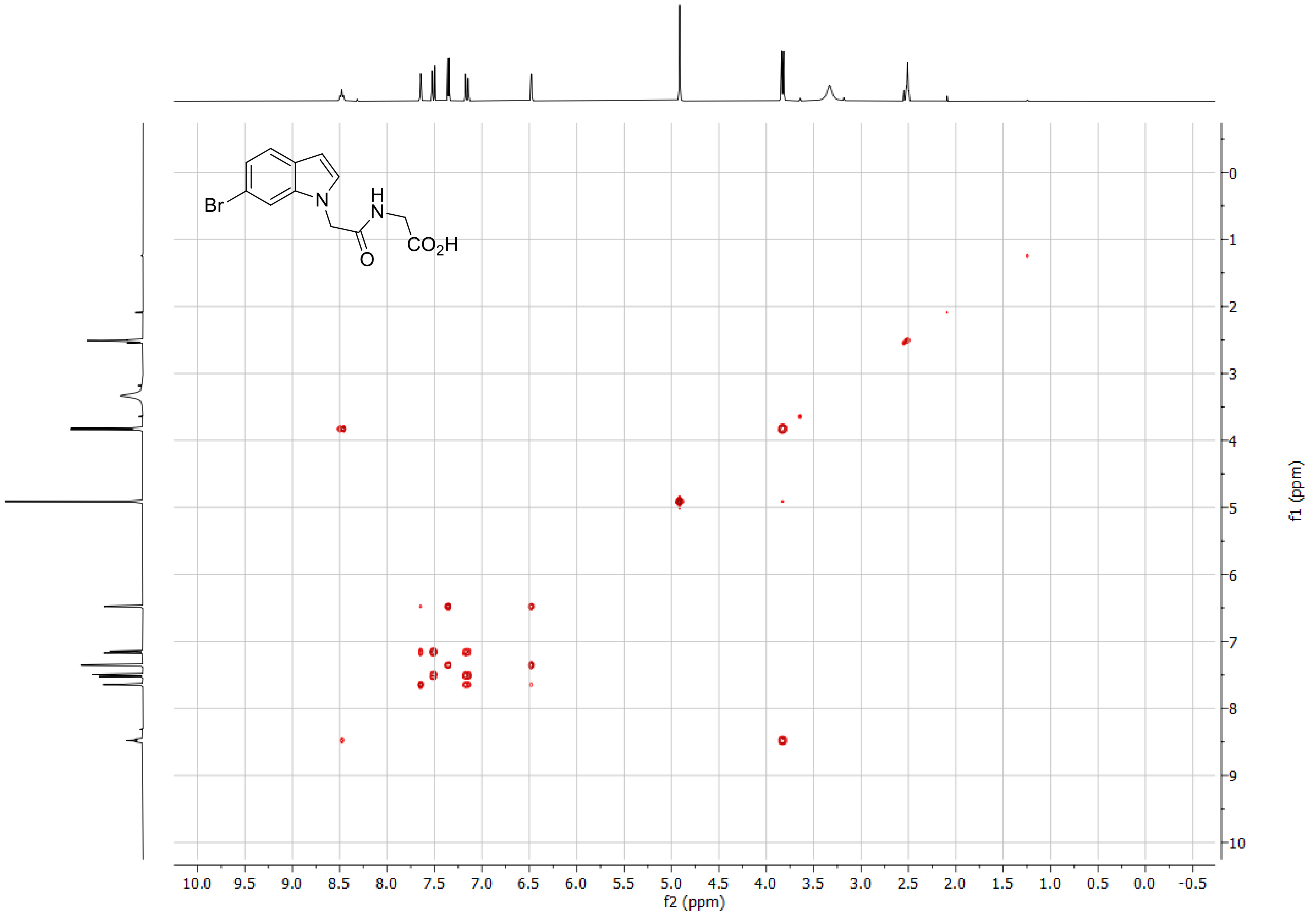

$^1H,^{13}C$-HSQC (400 & 101 MHz) in $CDCl_3$

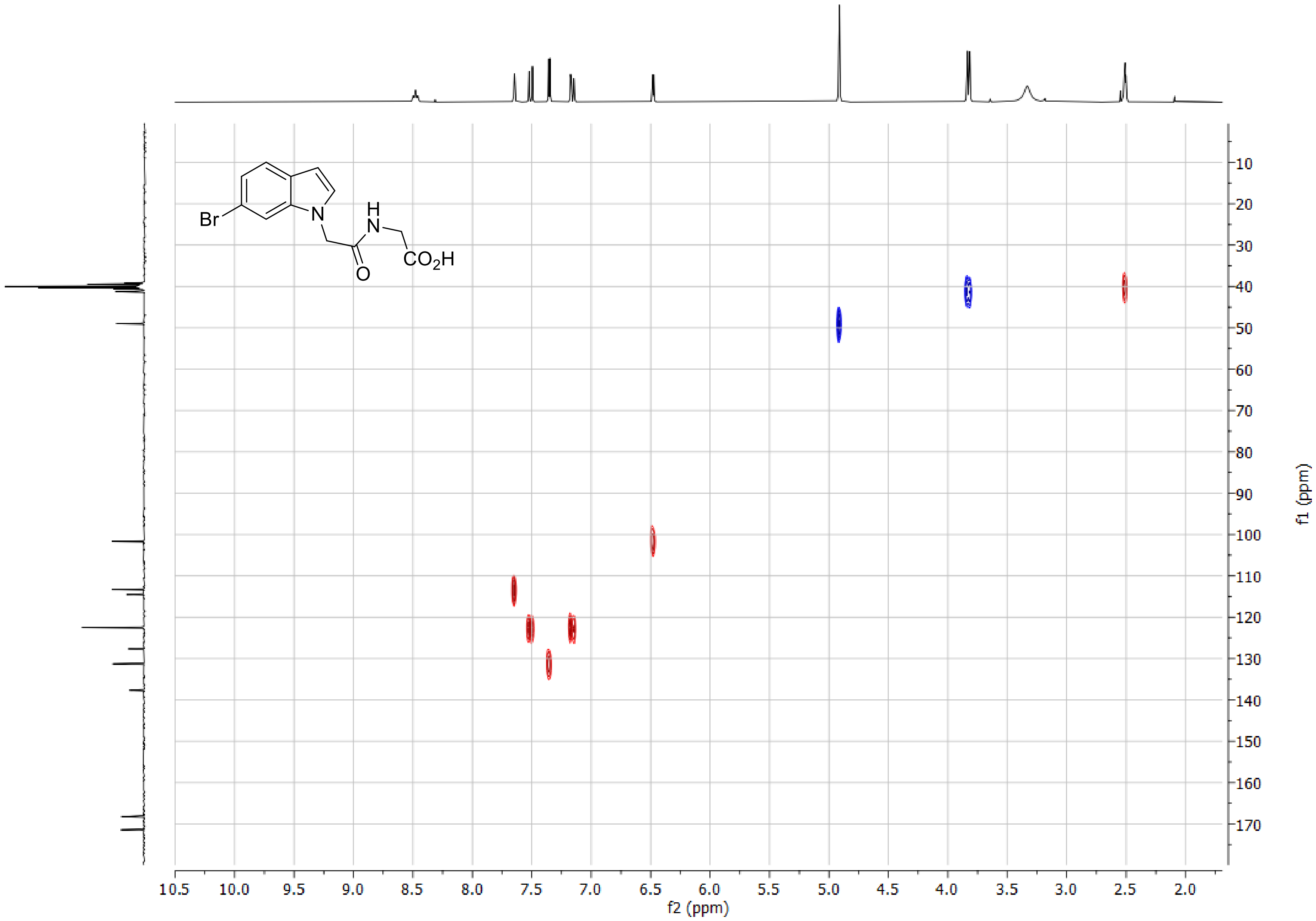

$^{1}H,^{13}C$-HMBC (400 & 101 MHz) in $CDCl_3$

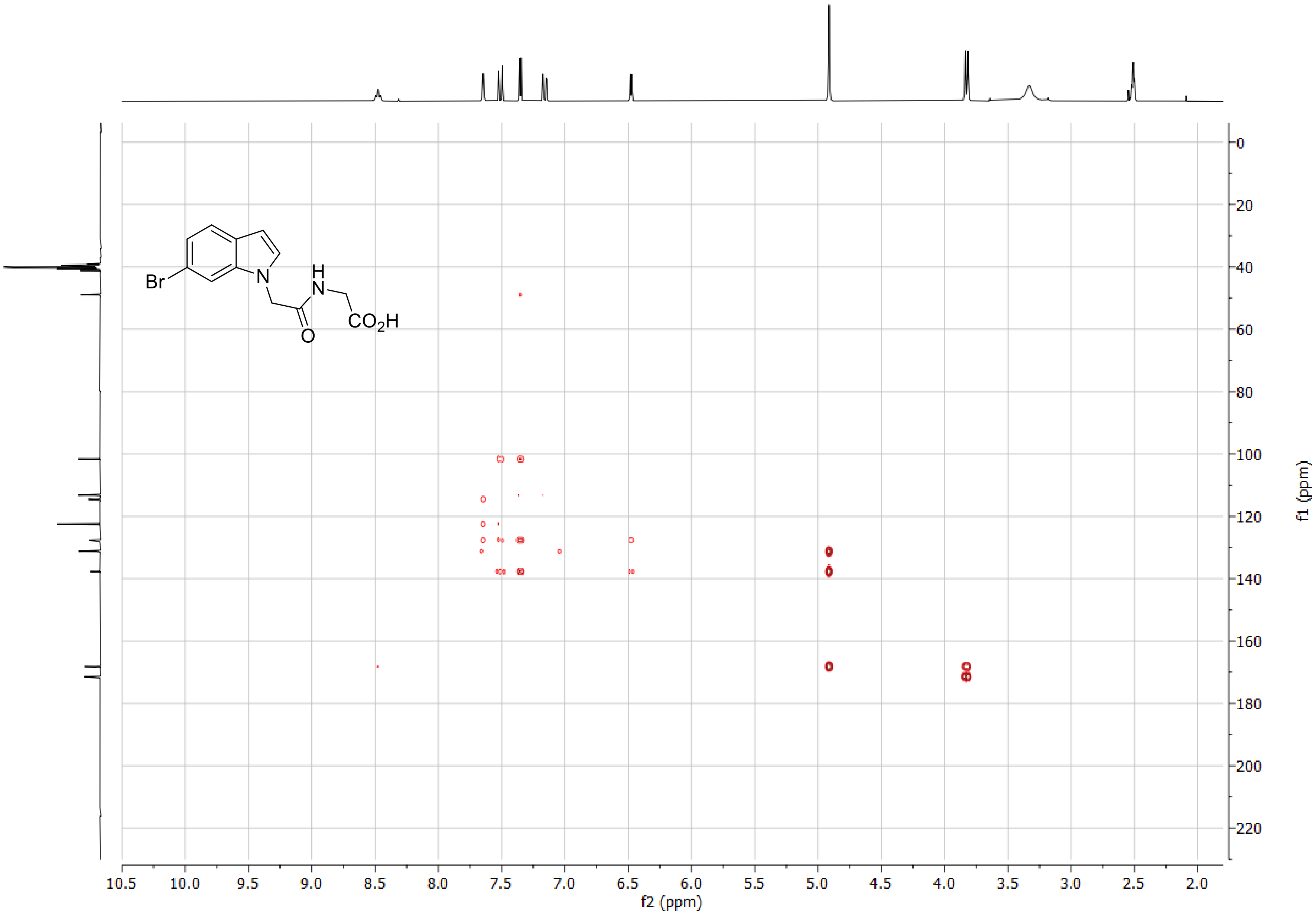

^{1}H NMR (300 MHz) in $CDCl_3$

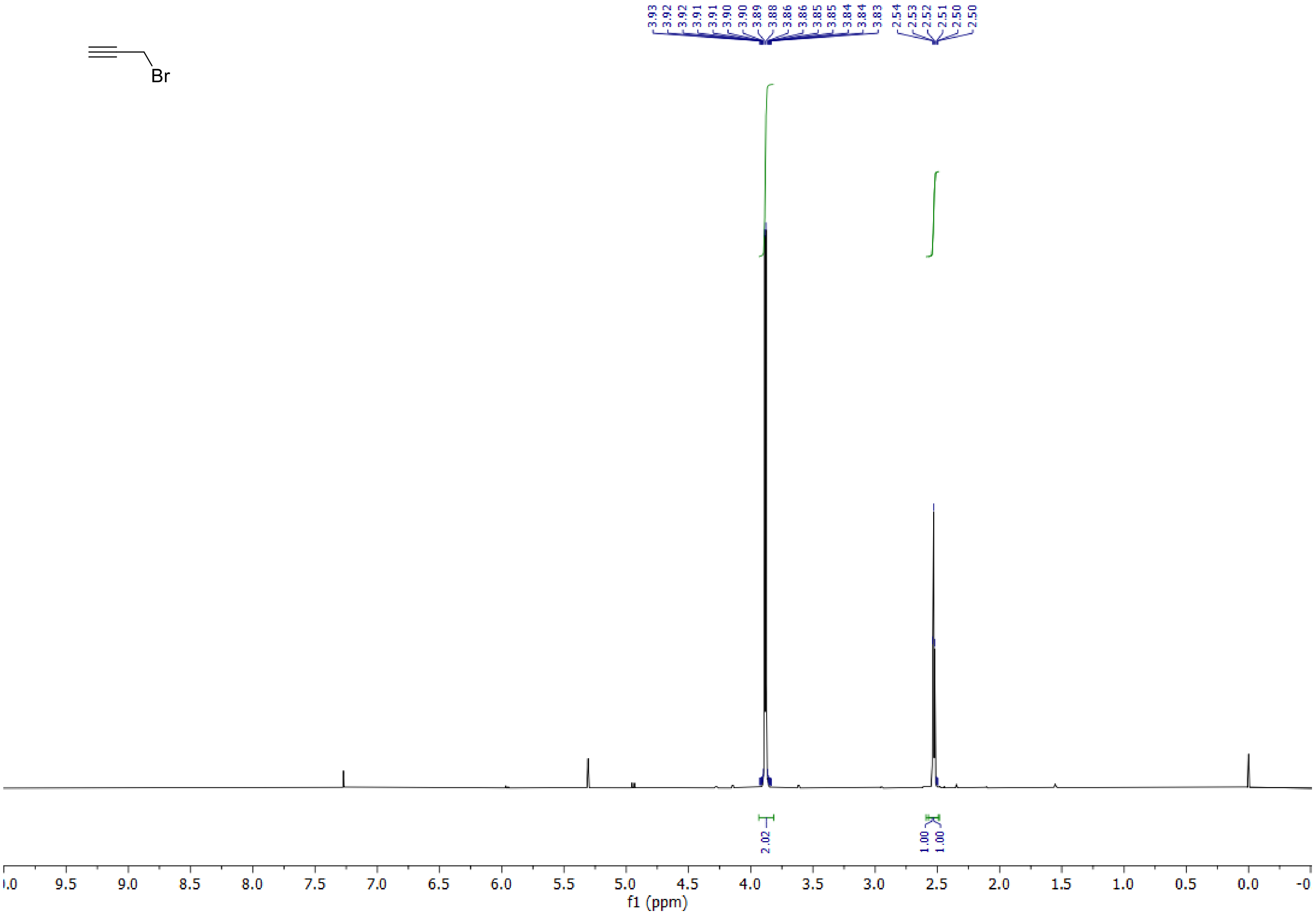

^{1}H NMR (300 MHz) in $CDCl_3$

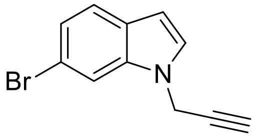

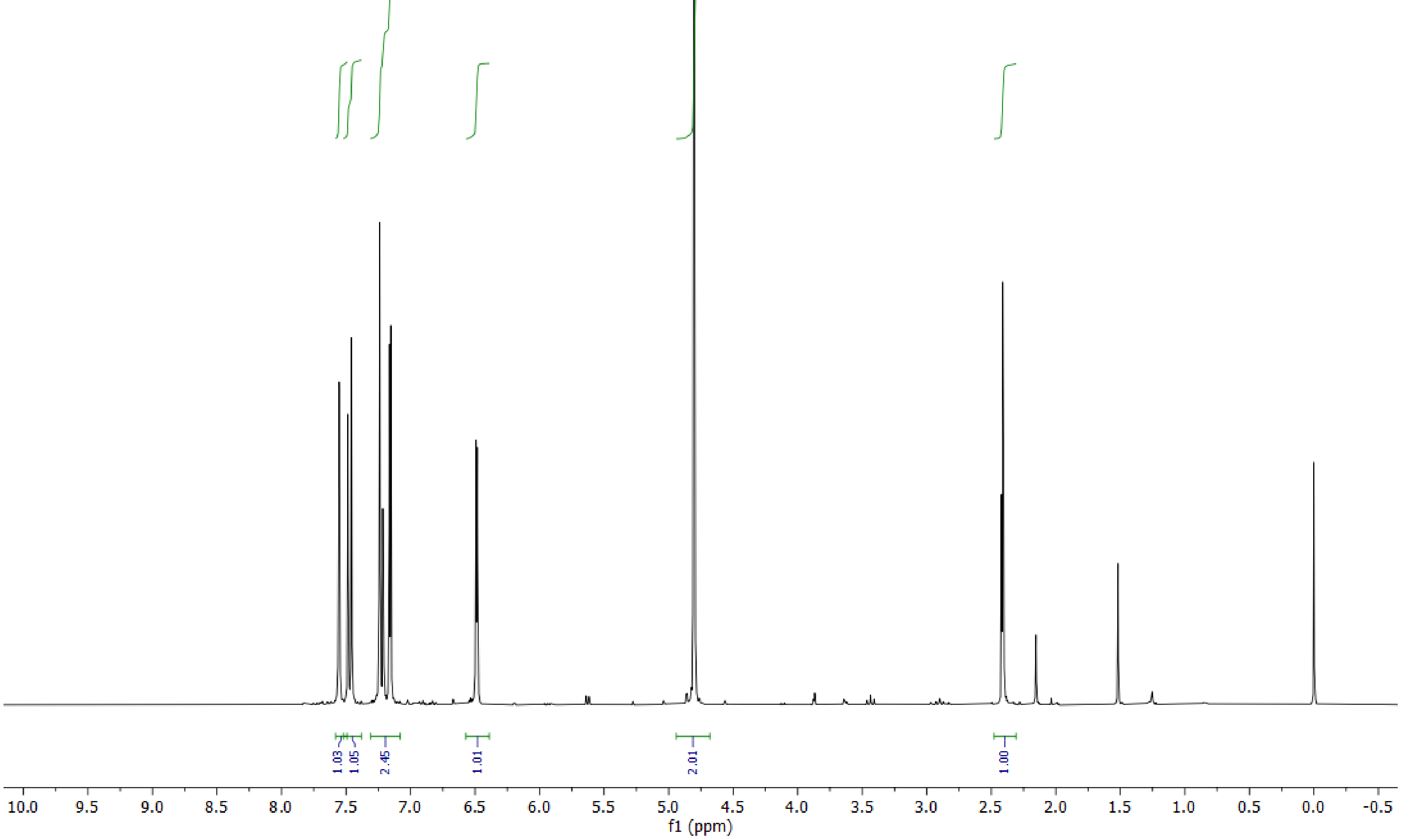

^{13}C NMR (75 MHz) in $CDCl_3$

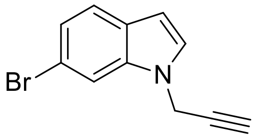

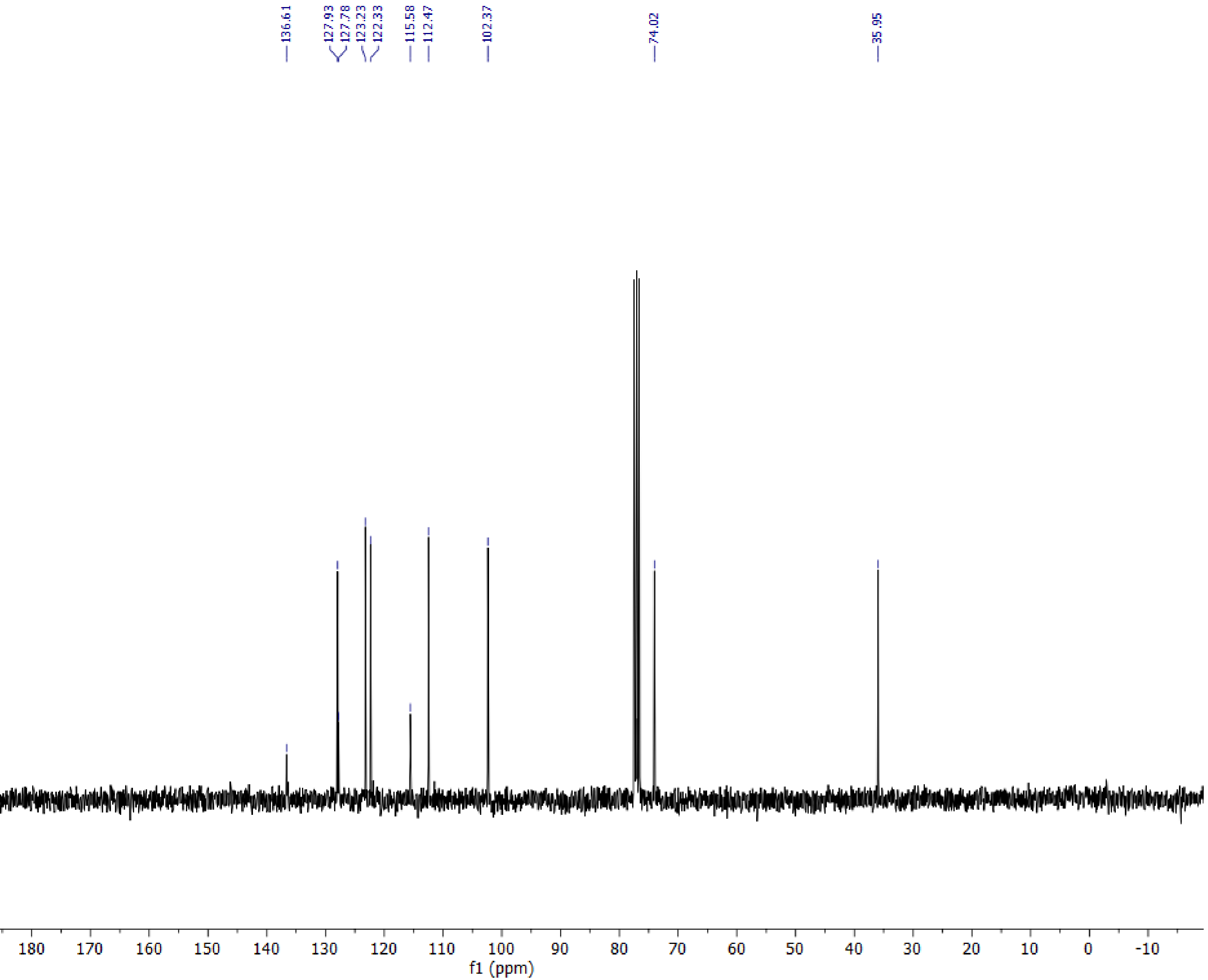

^{1}H NMR (300 MHz) in $CDCl_3$

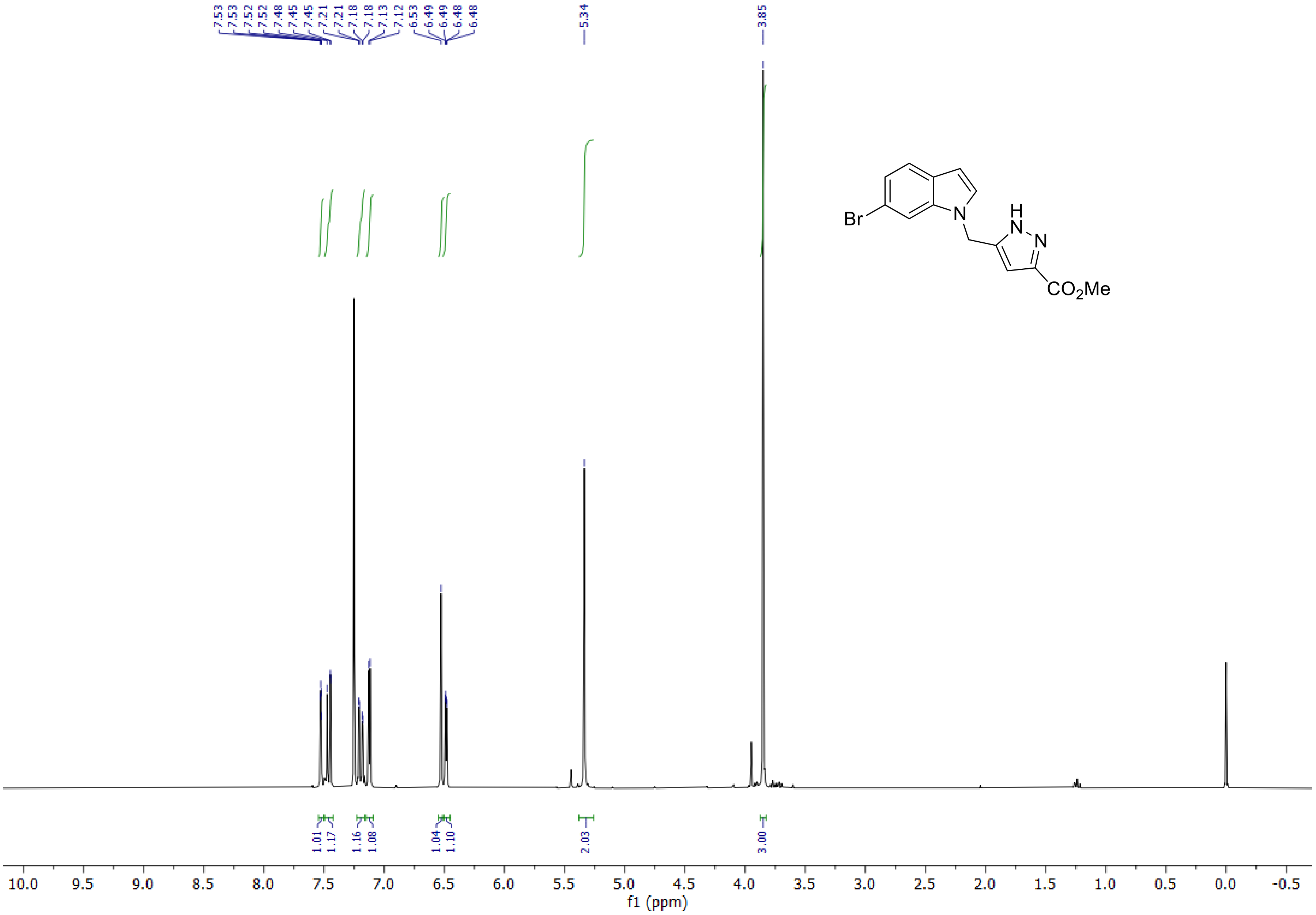

^{13}C NMR (75 MHz) in $CDCl_3$

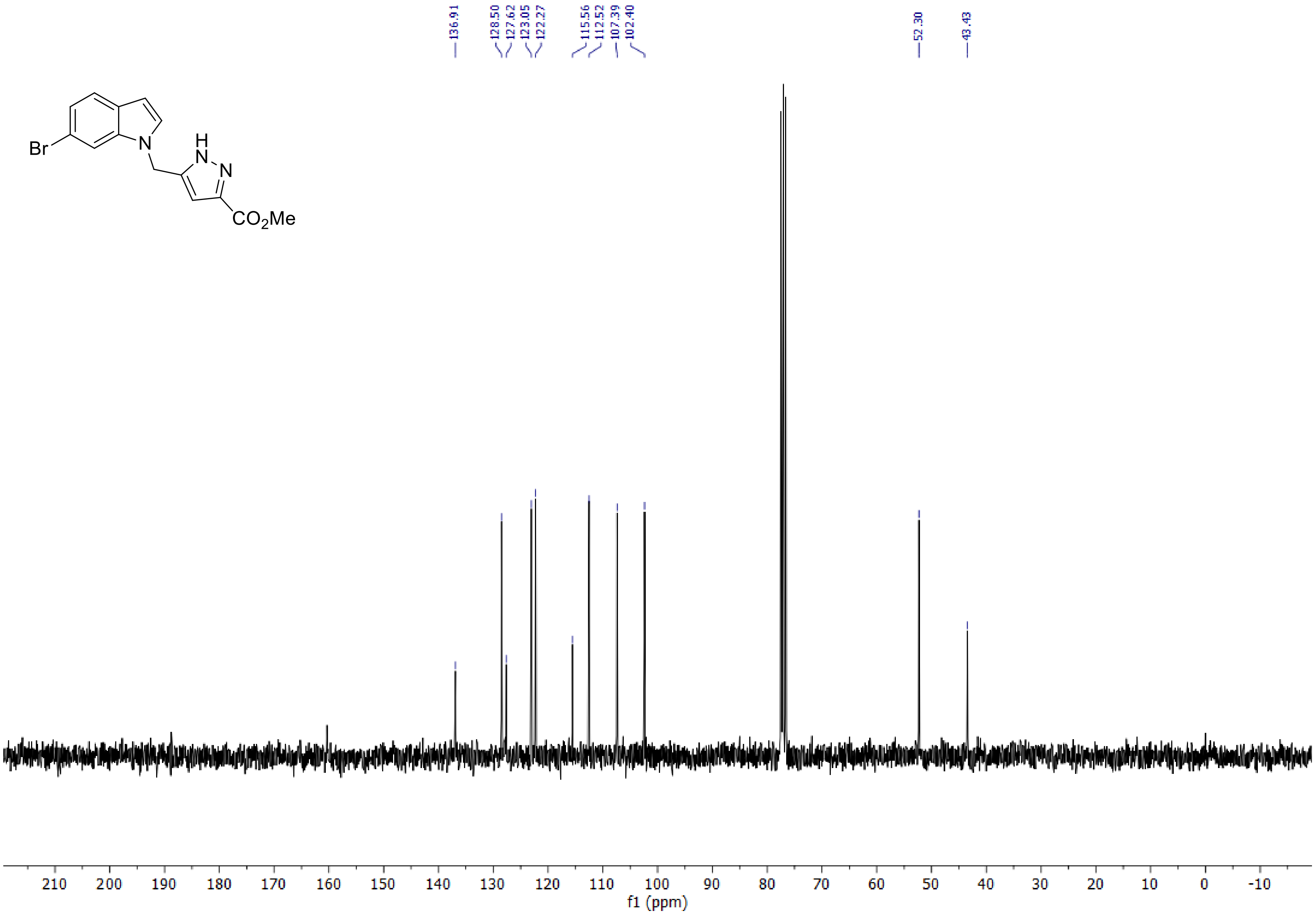

^{1}H NMR (300 MHz) in DMSO-d6

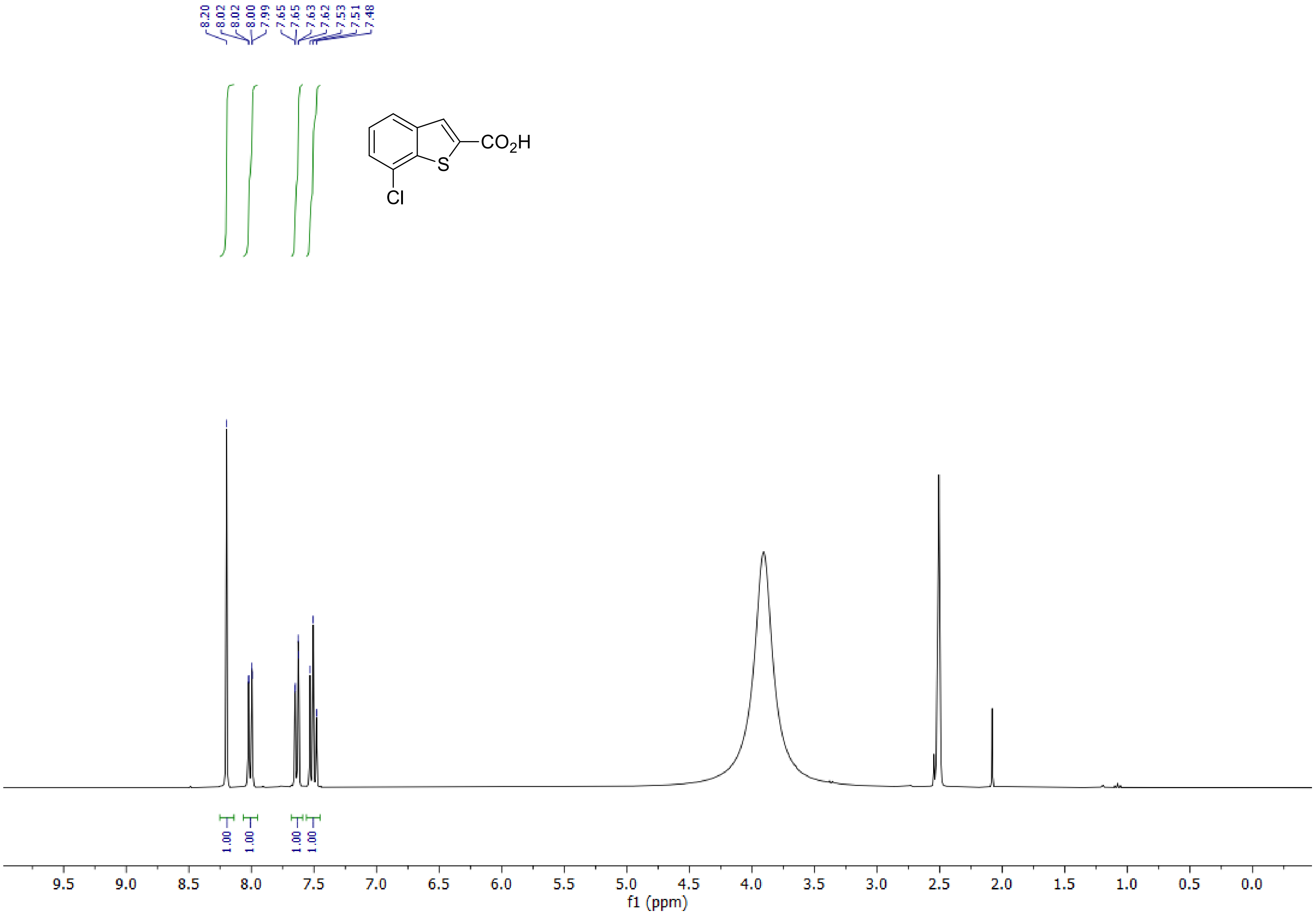

^{13}C NMR (75 MHz) in DMSO-d6

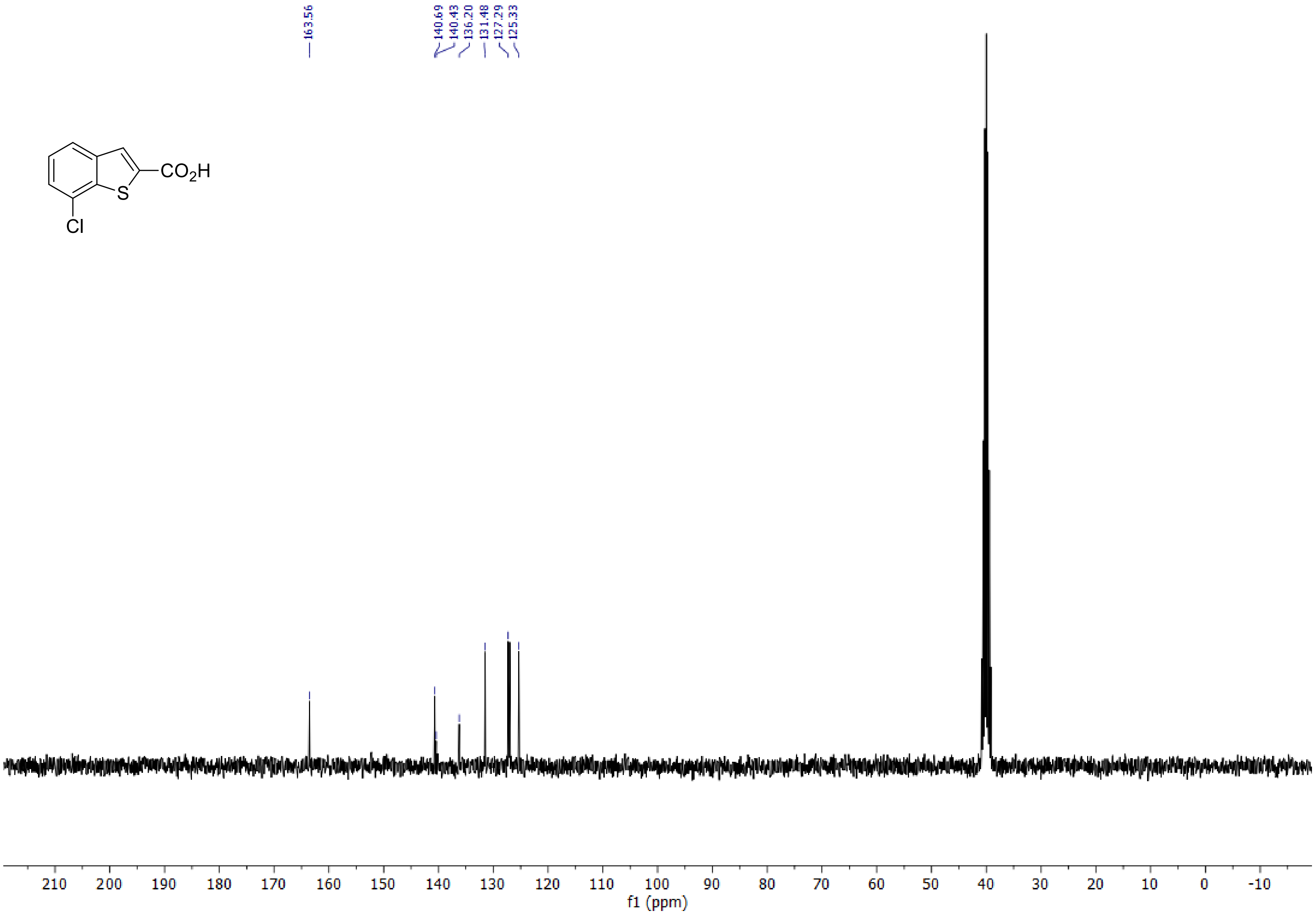

H NMR (300 MHz) in $CDCl_3$

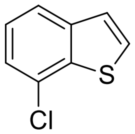

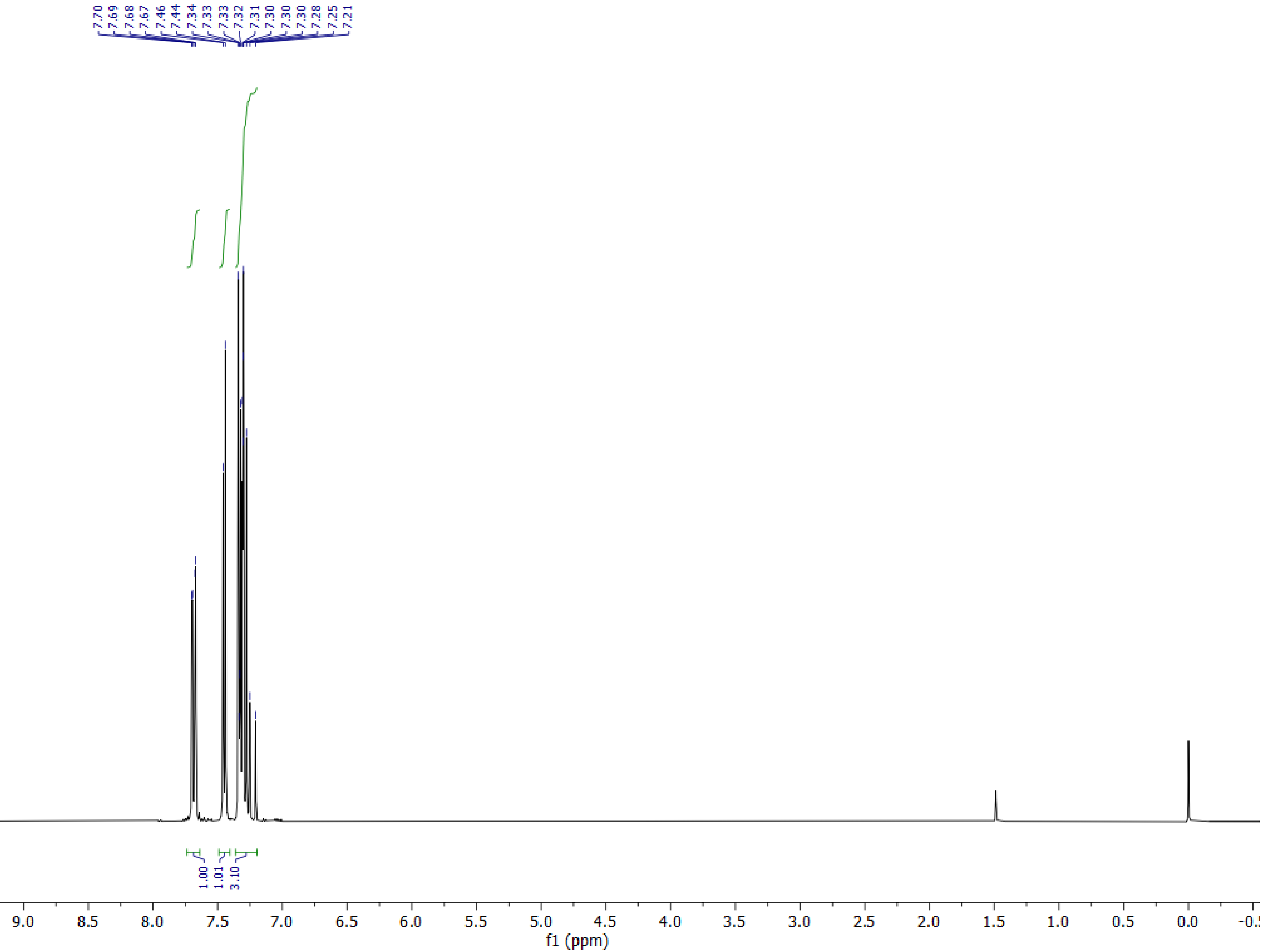

^{13}C NMR (75 MHz) in $CDCl_3$

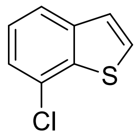

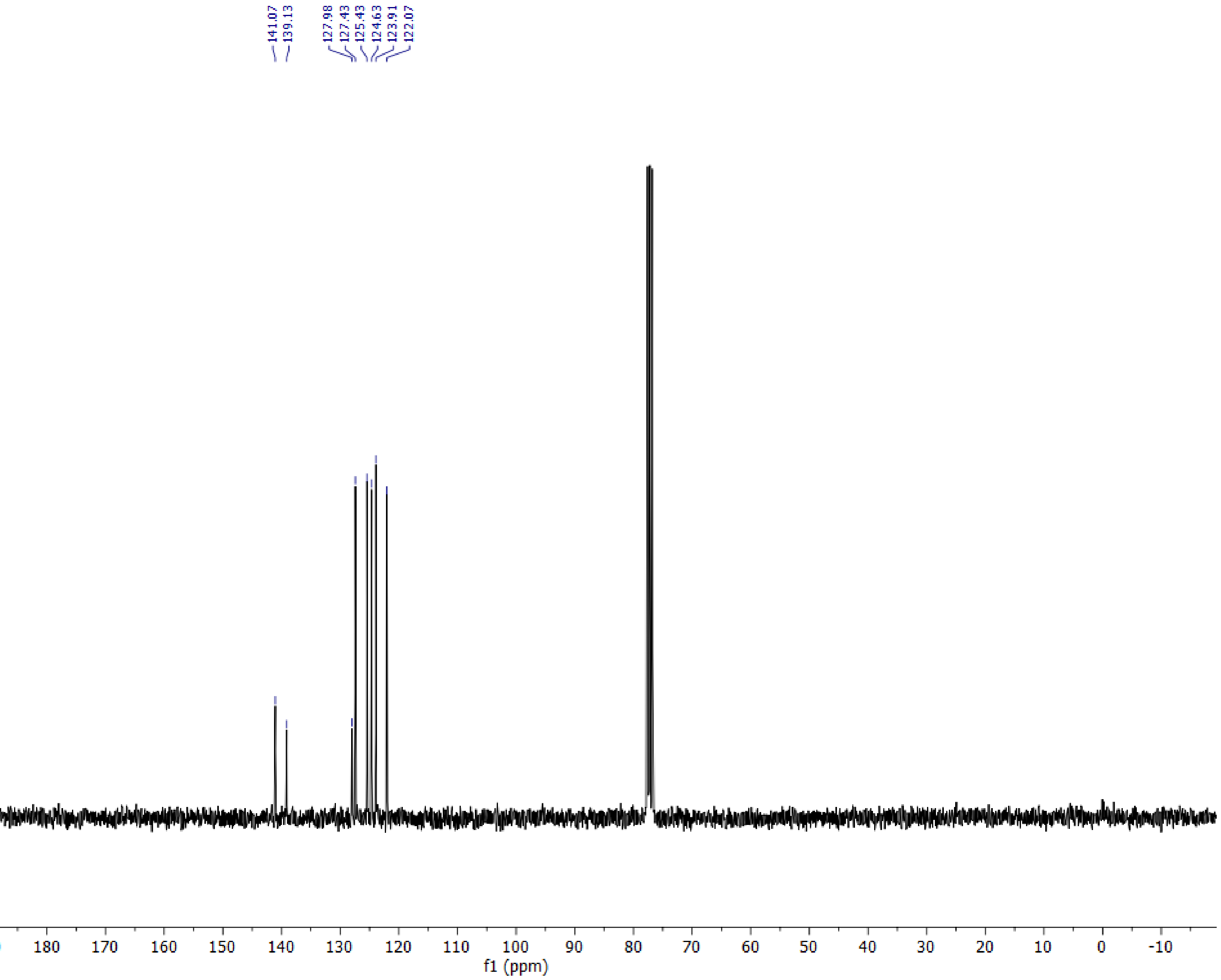

^{1}H NMR (300 MHz) in DMSO-d6

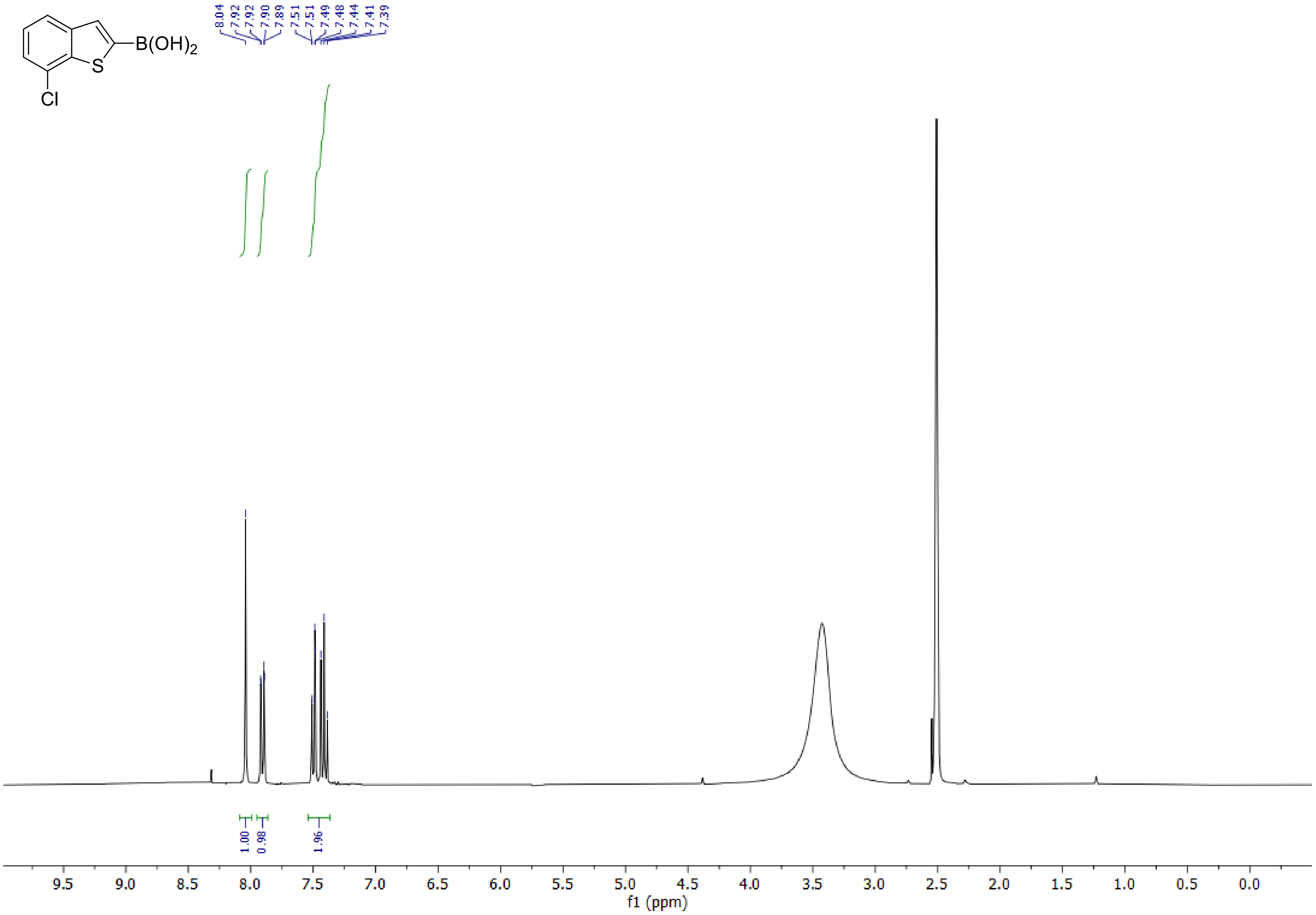

^{1}H NMR (300 MHz) in DMSO-d6

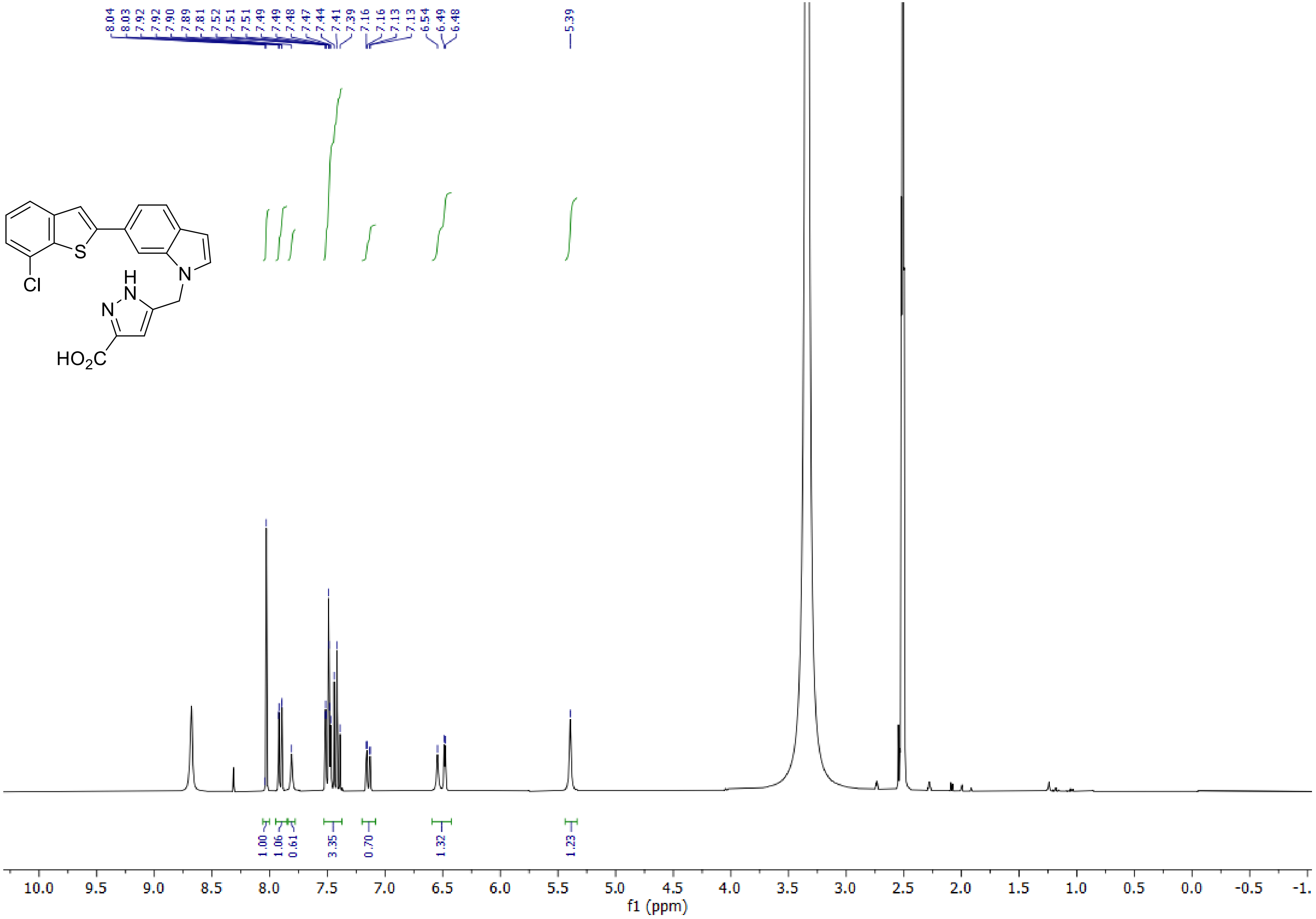

^{13}C NMR (75 MHz) in DMSO-d6

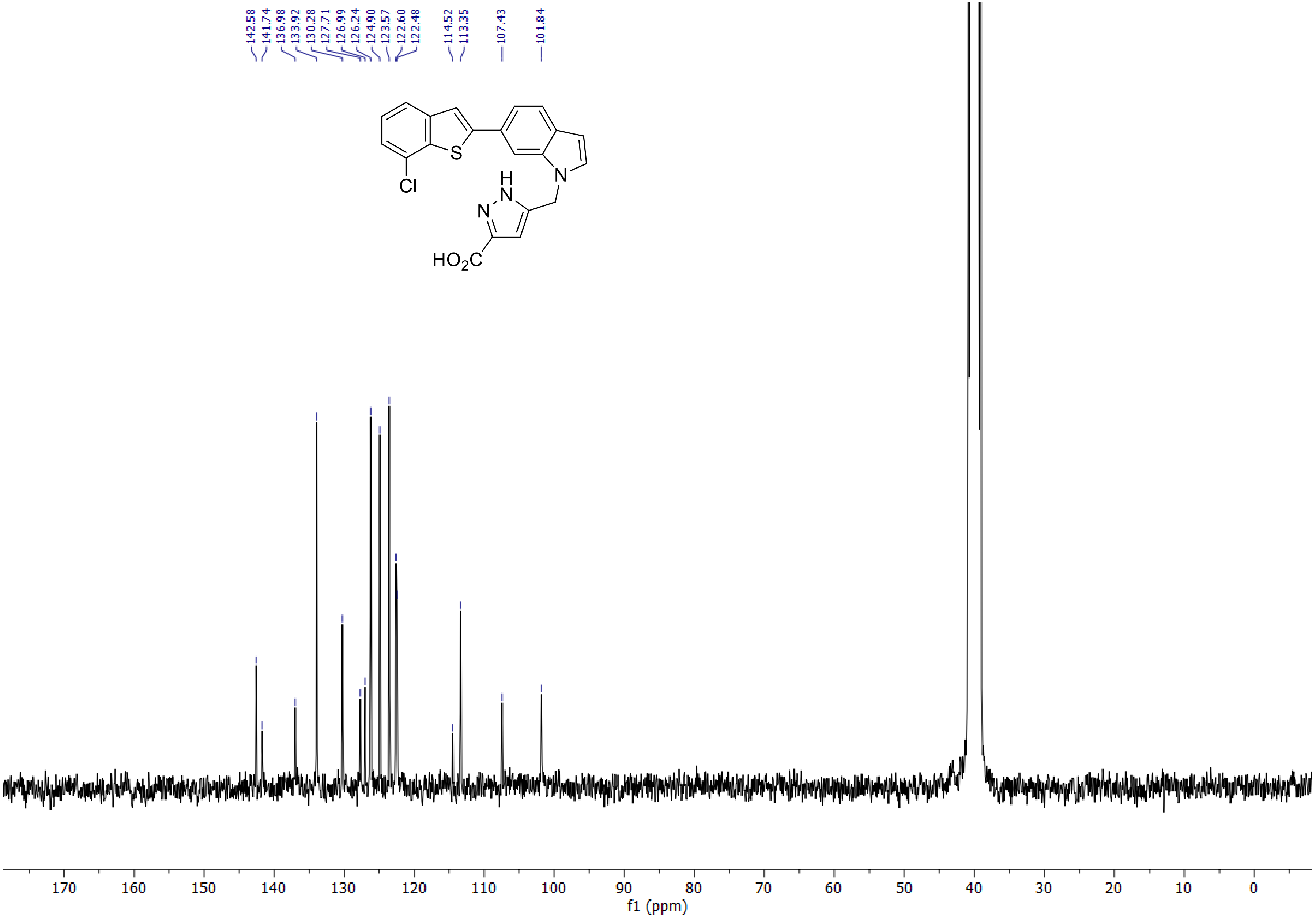

Supplement: Supplementary file 1 [file molecules-28-03568-s001.zip › molecules-2308393-SI.pdf]
